# Tissue Homeostasis in the Wing Disc of *Drosophila melanogaster*: Immediate Response to Massive Damage during Development

**DOI:** 10.1371/journal.pgen.1003446

**Published:** 2013-04-25

**Authors:** Salvador C. Herrera, Raquel Martín, Ginés Morata

**Affiliations:** Centro de Biología Molecular CSIC–UAM, Universidad Autónoma de Madrid, Madrid, Spain; Harvard Medical School, Howard Hughes Medical Institute, United States of America

## Abstract

All organisms have developed mechanisms to respond to organ or tissue damage that may appear during development or during the adult life. This process of regeneration is a major long-standing problem in Developmental Biology. We are using the *Drosophila melanogaster* wing imaginal disc to study the response to major damage inflicted during development. Using the Gal4/UAS/Gal80^TS^ conditional system, we have induced massive cell killing by forcing activity of the pro-apoptotic gene *hid* in two major regions of the disc as defined by Gal4 inserts in the genes *rotund (rn)* and *spalt (sal)*. The procedure ensures that at the end of a 40–48 hrs of ablation period the great majority of the cells of the original Rn or Sal domains have been eliminated. The results indicate that the damage provokes an immediate response aimed to keep the integrity of the epithelium and to repair the region under ablation. This includes an increase in cell proliferation to compensate for the cell loss and the replacement of the dead cells by others from outside of the damaged area. The response is almost contemporaneous with the damage, so that at the end of the ablation period the targeted region is already reconstructed. We find that the proliferative response is largely systemic, as the number of cells in division increases all over the disc. Furthermore, our results indicate that the Dpp and Wg pathways are not specifically involved in the regenerative response, but that activity of the JNK pathway is necessary both inside and outside the ablated domain for its reconstruction.

## Author Summary

The study of how organs or tissues regenerate after damage is a classic topic in Developmental Biology. We are studying this process in the developing wing imaginal disc of *Drosophila melanogaster*, using genetic methods to inflict massive damage in the region destined to form the wing blade. We find that the lesion provokes a very strong and rapid reaction in the remaining disc aimed to reconstruct the lost tissue, both in size and in shape. The response includes an increase of cell proliferation to compensate for the loss of cells and the immigration of cells from neighbouring areas to replace the dead ones. The immigrant cells change their original identity and acquire that of the cells they are replacing. We propose that these experiments reveal the existence of a powerful homeostatic mechanism that is able to cure massive injuries that may appear during development.

## Introduction

During the development or during the adult life organisms may suffer different kinds of insults, ranging from minor injuries to massive tissue damage or physical amputations. In consequence they have developed response mechanisms to reconstruct the damaged tissue. The phenomenon of regeneration is a major long -standing topic in Developmental Biology [1].

A principal factor in regeneration is the induction of the additional cell proliferation necessary to compensate for the lost tissue. Moreover, the new cells have to integrate with the pre-extant ones within the frame of the regenerating organ. Ultimately, regeneration is a matter of tissue homeostasis; it requires cooperative interactions between the old and the new parts of the tissue so that at the end the damaged organ is fully reconstructed in size and shape. This already points to the existence of overall control mechanisms that orchestrate the interactions of the different cell populations.

In *Drosophila melanogaster*, studies on regeneration have been carried out principally in the imaginal discs, the precursors of adult cuticular structures. These are named after the adult structure for which they are determined, wing disc, leg disc, eye-antennal disc and so forth. The imaginal discs are well-defined developmental systems formed by small groups of progenitor cells - ranging 10-50, depending on the disc [2]. They grow during the larval period, isolated from the larval tissues, and eventually differentiate sets of specific adult derivatives in stereotyped patterns. The imaginal discs are classical objects in developmental biology as they establish a clear distinction between determination and differentiation [3]. One important advantage of the imaginal discs is that after many years of study their development is very well known. Not only the basic growth parameters, but also the relevant developmental genes and signalling pathways have been described in detail [2], [4]

Our work has focused on regeneration in the wing disc, which is arguably the best characterised. The initial and final number of cells, the length of the cell proliferation period, the evolution of the cell division rates during development, etc, are known very precisely (see [5] and references therein). Moreover, during development it undergoes a process of gradual determination by which different cell populations progressively adopt defined developmental fates. This process is visualised by the appearance of distinct cell linage blocks (compartments) associated with the localised expression of developmental genes such *engrailed (en)*, *hedgehog (hh)*, *decapentaplegic (dpp*), *apterous* (*ap*), *wingless (wg)* and several others of more restricted expression.

At the end of the growth period the wing disc cells have attained a high degree of determination. This was demonstrated by experiments of transplanting disc fragments from fully developed discs into larvae close to metamorphosis, thus precluding further growth of the transplant. The transplanted fragments undergo metamorphosis along with the host larva and differentiate the adult patterns for which they were determined. These experiments showed that the various regions of the discs were strictly committed to differentiate specific adult patterns, such that it was possible to build maps of the presumptive adult structures, called “fate maps”, in the mature disc prior to differentiation [6]. This strict regionalization correlates with the localised expression in the mature disc of developmental genes such *pannier* [7], *vestigial*, *Distal-less*, *optomoter-blind*, *spalt* [8], *cut*, *achaete/scute* [2] that determine the differentiation and pattern of the adult structures.

Classically, the regenerative potential of the imaginal discs was assayed by transplanting fragments from mature discs into adult hosts, which permitted almost indefinite growth (*Hadorn and Buck, 1962*). These experiments showed that fully determined discs possess a high regenerative potential: a disc fragment can, under appropriate circumstances, regenerate the rest of the disc [9]. Work, especially from the Schubiger laboratory [10], [11] has established that ectopic activation of the *wg* gene is a major player in the process. Interestingly, the regeneration of *Hydra* head after amputation involves ectopic activation of the *wg* homologue Wnt in the region close to the cut [12]. Moreover, the regeneration of the zebrafish tail fin is also associated with Wnt/ß-catenin signalling, mediated by the *Wnt10a* and *Wnt5a* ligands [13].

The introduction of new methods of tissue ablation has provided a convenient approach to study the response of *Drosophila* discs to massive damage [14]. These methods rely on employing the Gal4/UAS system to force expression of pro-apoptotic genes that cause cell killing in specific disc domains. The usage of the temperature-sensitive form of the Gal4 -dominant suppressor Gal80 allows manipulation of the activity of the pro-apoptotic genes. It permits the control of the ablation time and the recovery process.

The work of Smith-Bolton et al., 2009 has established that after ablation of a large part of a growing disc, it is able to regenerate the damaged tissue to form normal adult structures. They find increased proliferation in the region close to the injury, associated with ectopic expression of the *wg* and *dMyc* genes in the damaged tissue. The activities of the Jun-N terminal Kinase (JNK) and the Hippo pathway are also required for regenerative growth [15], [16], [17].

Considered together, all the preceding experiments suggest a mode of regeneration in which the damage elicits a local response characterised by the activation of Wg/Wnt signalling pathways and associated with increased levels of cell proliferation.

However, there are examples of full regeneration in which there is no evidence of activation of signalling pathways. After massive cell death induced by irradiation, the *Drosophila* wing disc is able to compensate for the loss of cells and differentiates adult structures of normal size and shape. This phenomenon is known as compensatory proliferation [9] and, unlike the experiments cited above, it does not depend on *wg* activation [18]. This suggests that there may be alternative mechanisms to respond to damage.

Using a procedure as in Smith-Bolton et al., 2009, we describe the response of the wing imaginal disc to the ablation of two regions, as defined by the expression domains of the genes *rotund* (*rn*) [19] and *spalt* (*sal*) [20]. We find that the damage provokes a very rapid response of cells from outside the ablated tissue. These cells immigrate into the damaged territory and maintain the continuity of the epithelium and the genetic identity of the affected domain during the ablation time. These experiments reveal a very powerful homeostatic mechanism that is able to repair damaged domains almost contemporaneously with the damaging process.

Unlike previous reports [14], [15] we find that the proliferative response after localised damage is largely systemic, as all regions contribute to the regeneration by increasing the number of cells in division. This suggests the existence of an overall control mechanism that measures the cell population and triggers the regenerative response. Our results also indicate that the JNK pathway is involved in the repair process, but that the Dpp and Wg pathways do not play a specific role.

## Results

### Experimental design

We targeted for massive cell killing two regions of the wing disc, defined by the *rotund*-Gal4 and *sal^EPv^*-Gal4 lines. The Rotund (Rn) domain covers most of the wing pouch, approximately 40% of the entire disc. The Sal^EPv^ domain is smaller, occupies the central region of the wing pouch and represents about 16% of the disc (Figure S1). These lines were also used in previous studies [14], [15].

We paid special attention to the choice of the pro-apoptotic vectors. It was important to develop a procedure of cell killing that is very effective and also that allows a precise definition of the chosen domain, so that the damage is restricted to the target area. We tested six different UAS vectors, directing the expression of *head involution defective* (*hid*), *reaper* (*rpr)*, *Drosophila inhibitor of apoptosis1*-*RNAi (diap1-IR)*, *eiger (egr)*, *Drosophila homolog of p53 (dp53)* and the activated form of *hemipterous* (*hep^CA^*). We find that the damage inflicted by these pro-apoptotic vectors can be quite different, depending on the vector (details of the comparison are provided in Figures S2 and S7). A particularly important aspect is the capacity of the disc to regenerate fully after the treatment with the pro-apoptotic vector. In the standard conditions used in our experiments, 40–48 hrs of ablation, the UAS-hid construct allows full regeneration of the disc, whereas after treatment with the other vectors the regeneration is incomplete (Figure S2). Consequently, we have used the UAS-hid vector in our experiments. The fact that different vectors may cause distinct kinds of damage raises the possibility of different regenerative responses (see below). A summary of the comparison of the various vectors is presented in Table S1

To induce massive but conditional cell death in the Rn domain we constructed larvae of genotype *rn-Gal4 tub-Gal80^TS^ UAS-hid UAS-Flp act>stop>lacZ* (see Exp. Proc. for details). In this genotype there is no significant apoptosis in the wing disc at 17°C, (permissive temperature for Gal80^TS^) but massive apoptotic levels at 29°C (restrictive temperature) in the Rn domain. In addition, the UAS-Flp transgene will be activated to produce large amounts of Flipase that will recombine the *act<stop<lacZ* cassette in the cells of the domain. As a result all or nearly all the cells of the target domain are indelibly labelled by *lacZ* activity (Figure S3). This system is very similar to that utilised by Smith-Bolton et al., 2009. To induce cell death in the Sal^EPv^ domain we used a similar genotype, just replacing the rn-Gal4 line for sal^EPv^-Gal4.

### Ablation experiments

We first tested the efficacy of the method by allowing continuous activity of Hid in the Rn and Sal domains, achieved by rearing the *rn-Gal4 tub-Gal80^TS^ UAS-hid UAS-Flp act>stop>lacZ* and *sal^EPv^-Gal4 tub-Gal80^TS^ UAS-hid UAS-Flp act>stop>lacZ* animals all the time at 29°C. This results in adult flies in which the regions corresponding to either the Rn or the Sal^EPv^ domain are lacking (Figure S1).

In the conditional ablation experiments temperature shifts (17 to 29°C and vice versa) were used to control the stage and the timing of the cell killing process. The standard protocol was to allow normal larval growth (17°C) until day 7 after egg-laying, which corresponds to interface between the second and the third larval period. Then the larvae were shifted to 29°C for various lengths of time before been shifted back to 17°C.

The first experiments were aimed to find out the maximal ablation time that was compatible with full reconstruction of the targeted domain. The results are illustrated in Figure S1. Treatments of 48 hrs at 29°C still allow nearly full recovery of the size and pattern of the wing. After 72 hrs of Hid activity there is some recovery but the wings are smaller than normal and show pattern defects. Consequently, in the majority of the experiments the ablation time was of 40–48 hrs.

To follow the reconstruction process, the discs were fixed at different times after the initiation and the end of the treatment. As indicated above, the experimental system allows the identification of the cells of the ablated domain. We checked the efficacy of the marking system in control experiments in which the *UAS-hid* construct was substituted by *UAS-GFP*. In this case the temperature shift does not produce cell killing, but the activity of the *UAS-Flp* construct will induce flip out of the *act<stop<lacZ* cassette thus marking the cells of the Rn domain. The result of this experiment is that all the cells of the domain become marked with *lacZ* activity (Figure S3). This is a significant result, for it indicates that in the experimental series we can mark all the original cells of the ablated domain and therefore we can trace the lineage of the reconstructed tissue.

One important parameter to estimate was the percentage of cells of the domain killed in the experiments. In our experiments we find a co-extensive expression of caspase-positive and *lacZ*-expressing cells (Figure S3), suggesting that we are eliminating the great majority of the cells of the domain. Nevertheless, this estimate is problematic because, as we discuss below, there is a continuous immigration of cells into the domain during the ablating procedure. A careful counting of cells marked for caspase and Topro after 24 hrs of Hid activity indicates a 55% of cell killing in the Rn domain. It suggests that after 48 hrs of ablating time most of the original cells of the domain have been killed.

### Evolution of the ablated domains

**Figure 1:**
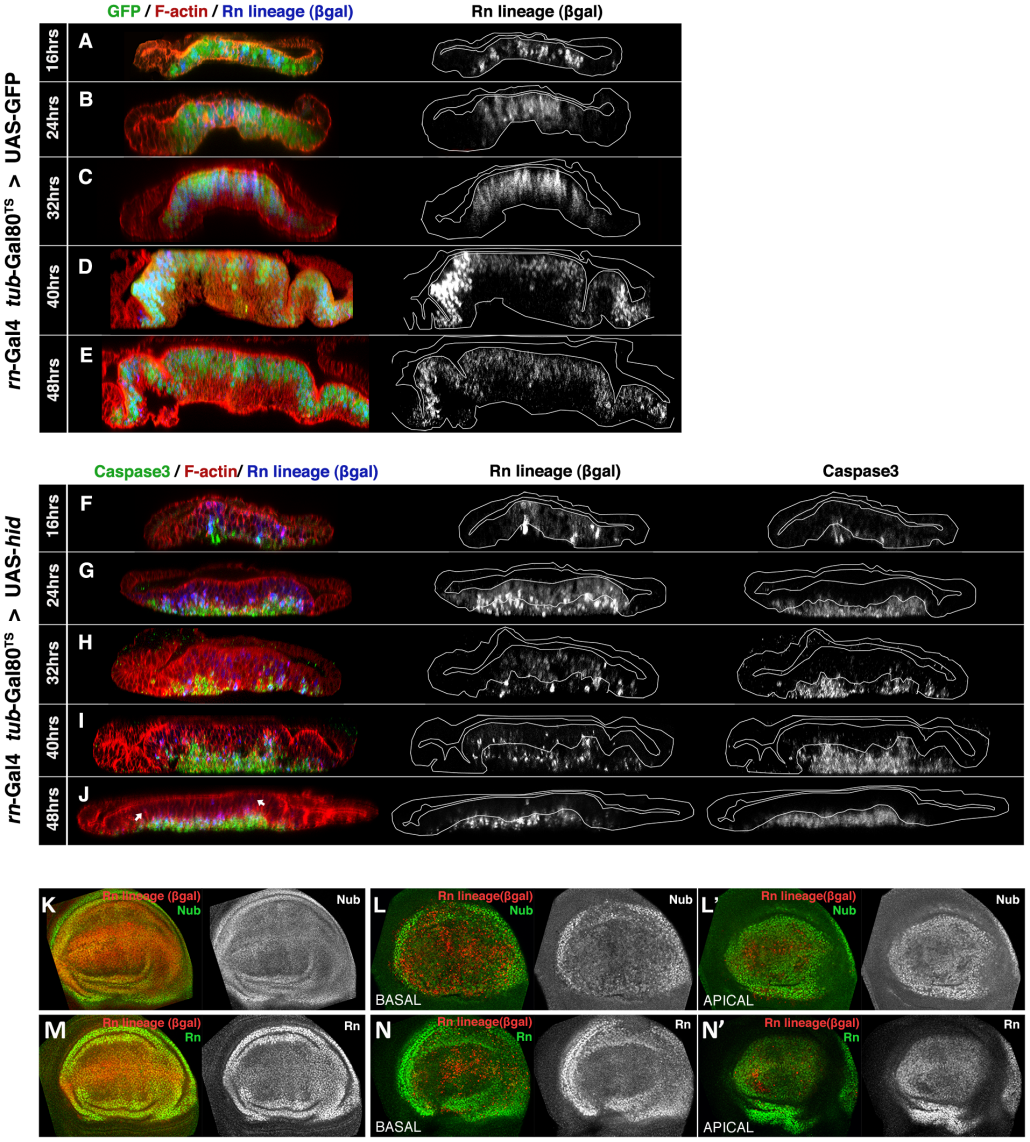
Rn domain reconstruction during ablation. (A–J) Orthogonal sections of wing discs perpendicular to the A/P border (at the maximum width of the rotund domain) of genotypes *UAS-GFP/tub-Gal80^TS^; rn-Gal4/UAS-Flp act>stop>LacZ* (control, A–E) and *UAS-hid tub-Gal80^TS^/+; rn-Gal4/UAS-Flp act>stop>LacZ* (experimental, F–J). The temperature shift (17 to 29°C, TS) was given at day 7 of development. Times after the TS are indicated to the left. The outlines of living epithelium (excluding dead cells) are indicated with white lines. Peripodial membranes (were neither *rn* nor *sal* are expressed) are located at the top of the panels. In control discs (A–E), 24 hrs after the TS all the cells of the Rn domain are already labelled both by GFP and ßgal. The ßgal staining represents the lineage of the cells, in which the flip out of the *act>stop>LacZ* casette marks their progeny indelibly with *lacZ* expression. Note the near perfect coincidence between the Rn lineage and GFP expression. (F–J) The experimental discs were fixed at the end of various times of Hid-induced ablation, shown at the left side to the panels, without recovery time. 16 hrs after the TS (F) there are already some cells expressing lacZ, indicated by the ßgal staining. The few cells in apoptosis are extruding towards the basal surface. By 24 hrs after TS (G) most of the cells in the RN domain contain ßgal, while a number of apoptotic cells have been extruded to the basal section. Many of these Caspase 3 positive cells also contain ßgal. After 32, 40 and 48 hrs of Hid induction (H–J) the number of ßgal cells in the apical plane diminishes, as they enter apoptosis and are extruded basally (middle panels in I, J). These cells are gradually replaced by non-ßgal cells that originate from outside the original Rn domain. Note that despite the massive apoptosis, the immigrant non- ßgal cells maintain the continuity of the epithelium. (K–N) Wing discs of the previous genotypes at the end of 48 hrs of GFP (K and M, controls) or Hid (L–L′, N–N′) induction, stained for the wing pouch determinants Nubbin (Nb) or Rotund (Rn). The distribution of the Nub and Rn proteins in the experimental discs at the end of the ablation period (L–N′) is very similar to that of the control (K, M). This suggests that at the end of the ablation process the tissue is largely repaired.

We have followed the evolution of the Rn domain during the ablation period and also after the end of it. Unexpectedly, it turned out that the most significant events take place contemporaneously with the massive cell killing. In Figure 1F-1J we illustrate the changes that occur during a 48 hrs Hid treatment in comparison with control discs in which *hid* is not activated (Figure 1A–1E). 16 hrs after the initiation there already is noticeable apoptosis and also many of the cells in the domain express *lacZ*. The cells in apoptosis are extruding towards the basal surface of the disc. At 24 hrs *lacZ*-expressing cells occupy most or all the domain but only a fraction of them are in apoptosis. Since both Flipase and Hid activities depend on Gal4, this suggests that flip-induced recombination takes shorter than Hid-induced apoptosis. Both in control (Figure 1B) and in experimental discs (Figure 1G), after 24 hrs of Flp activity, the great majority of the cells in the Rn domain are labelled with *lacZ*. After 32 hrs of Hid treatment many of the *lacZ*-expressing cells are disappearing and their remains accumulate in the basal surface. However, the apical section of the disc appears normal and contains numerous cells that do not express *lacZ*. This indicates that cells from outside the targeted domain (we refer to them as immigrant cells) are being incorporated. The process continues by 40 hrs, and finally by 48 hrs the Rn domain appears to be constituted by a majority of cells not labelled with *lacZ*. Its size is approximately 75% of the normal Rn domain.

We have assayed the genetic identity of the repaired domain at the end of the ablation period by examining the expression of *rn* and *nubbin (nub)*, genes that can be considered markers of the wing pouch identity [21], [22]. The result (Figure 1K–1N′) is that these genes are expressed as in a normal disc, indicating that the immigrant cells, which have repopulated the domain, have acquired the appropriate identity. The cellular debris, still containing caspase activity, accumulates basally.

There are three remarkable aspects of this process, i) during the ablation process there always is continuity in the epithelium, ii) at the end of the ablation process the Rn domain is almost completely reconstituted (Figure 1J). The expression of *rn* and *nub* also indicates a full genetic reconstruction (Figure 1L–1N′), iii) The Rn domain has been largely reconstructed by immigrant cells.

The results obtained in the experiment ablating the Sal^EPv^ domain reinforce the conclusions of the *rn>hid* experiment. At the end of 40 hrs of Hid activity the Sal^EPv^ domain appears largely or completely reconstructed; the apical surface is populated by a majority of immigrant cells, whereas the apoptotic debris accumulate in the basal surface (Figure S4)

**Figure 2:**
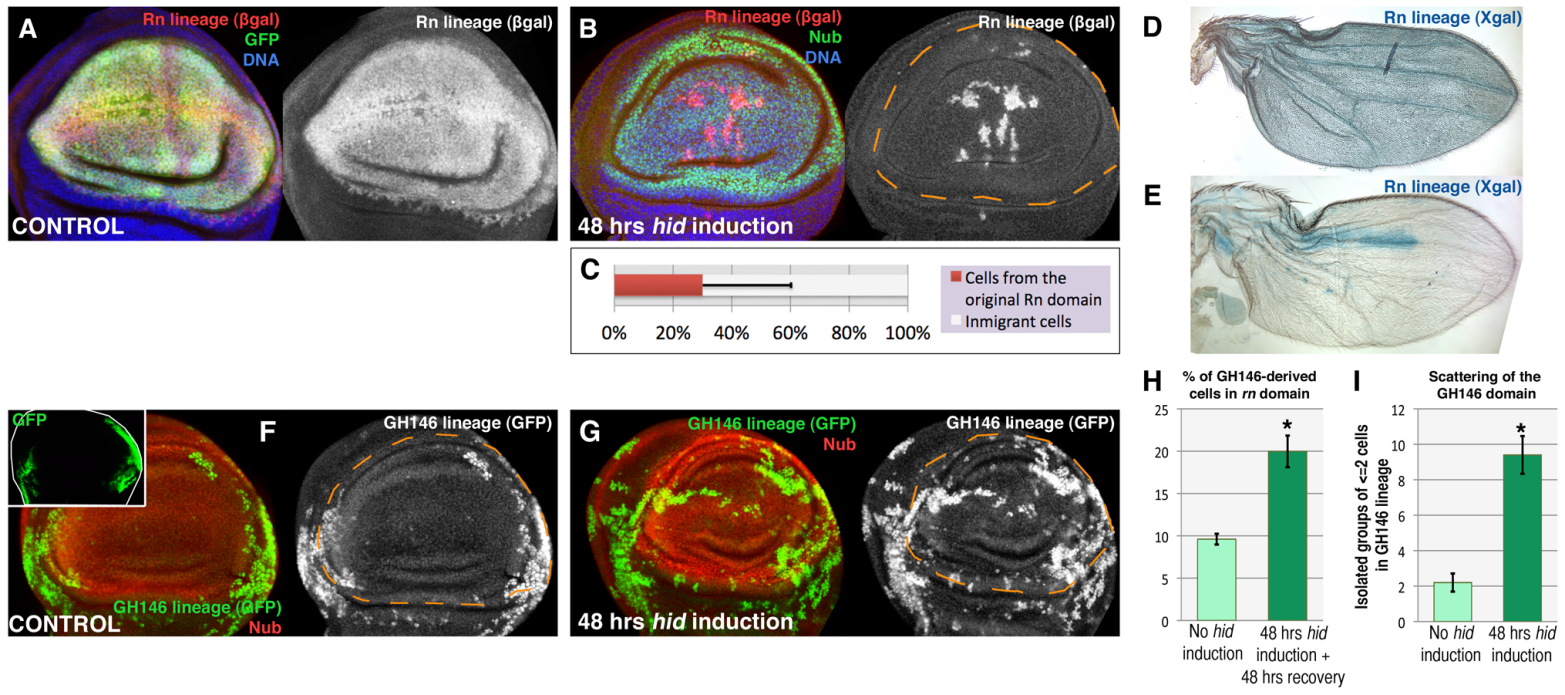
Reconstruction of the Rn domain by immigrant cells. (A) Rn lineage (white) of a control non-ablated disc of genotype *UAS-GFP/tub-Gal80^TS^; rn-Gal4/UAS-Flp act>stop>LacZ*. Note that all the Rn domain cells are labelled both by GPP and ßgal. B) Lineage of the Rn domain (white) of a disc of genotype *UAS-hid tub-Gal80^TS^/+; rn-Gal4/UAS-Flp act>stop>LacZ* fixed 48 hrs after a 48 hrs ablation period. To avoid the use of three UAS transgenes in the experiment we have marked the wing pouch with the anti-Nubbin antibody (in green, it covers a domain very similar to that of Rn). Notice that the greater part of the Nub domain is composed by cells that did not originate in the original Rn lineage as they do not contain ßgal activity. C) Average contribution of the original Rn domain to the final Nub domain (n = 20), which is about 30%. D, E) Visualization, using X-gal staining, of the contribution of the original Rn domain to adult wings (genotypes as in A and B). While in control flies X-gal staining covers the entire wing (D), in the experimental ones (E) it covers a small area. The wing portrayed in E is an extreme case in which about 90% of the territory is not marked with X-gal, indicating that the domain has been essentially reconstructed by immigrant cells. F–I) Making use of the QF/QUAS and the Gal4/UAS systems to mark independently cells of the wing hinge, defined by the GH146 QF line (inset), and the ablated region defined by the rn-Gal4 line. In control discs (F, H) the progeny of the cells of the GH146 (white) contributes little to the Nub domain, about 10% (shown in the light green bar in H, n = 16). In the experimental discs the GH146 lineage contributes to 20% of the Nub domain (G, H, dark green bar n = 17). (I) Scattering of the GH146 lineage, after 48 hrs of *hid*-expression (dark green bar, n = 20) compared with control (light green bar, n = 10), measured as the number of groups of 1 or 2 cells isolated from the rest of the lineage (n>12). The large number of isolated cells suggests the existence of cell migrations during the reconstruction of the domain. Error bars represent SD in panel C and SE in panels H and I (* = p<0,01).

We have estimated the contribution of immigrant cells to the repaired domain in discs fixed 48 hrs after the end of Hid treatment. At that time the Rn domain is fully normal. The *lacZ*-marked cells cover 25–30% of the repaired domain. Thus, the average contribution of immigrant cells is about 70% (Figure 2B, 2C). However, there are cases in which the non-*lacZ* territory is about 90% (Figure 2E), indicating that the domain can be almost entirely rebuilt by immigrant cells. The 70% value is probably an underestimate because some of the *LacZ* expressing cells may be late immigrants into the domain that performed the flip out, but in which the apoptotic program was not executed.

To explore further the possibility of the immigration of cells into the Rn domain we have used the QF/QUAS system [23] as an independent method to mark cells. The Q line GH146 is expressed predominantly in part of the hinge region of the wing disc (Figure 2), although there is some overlap with the rotund domain. The comparison of the contribution of the GH146 cells to the normal Rn domain or after massive cell killing (Figure 2F, 2G, 2H) clearly indicates that many cells originally in the hinge region contribute to the repaired tissue. Moreover, it has to be considered that only a fraction of hinge cells are marked with the GH146 line, thus the contribution of unmarked outside cells cannot be scored. We also observe that in the reconstructed domain many cells of the GH146 lineage appear to be scattered (Figure 2G). The amount of scattering in greater than in control discs (Figure 2I), suggesting the existence of cell movements during the reconstruction process.

### Cell proliferation during ablation: A systemic response

One of the principal features of the regeneration process is the induction of a proliferative response necessary to generate new cells [24], [25]. We have used several methods to measure this response.

**Figure 3:**
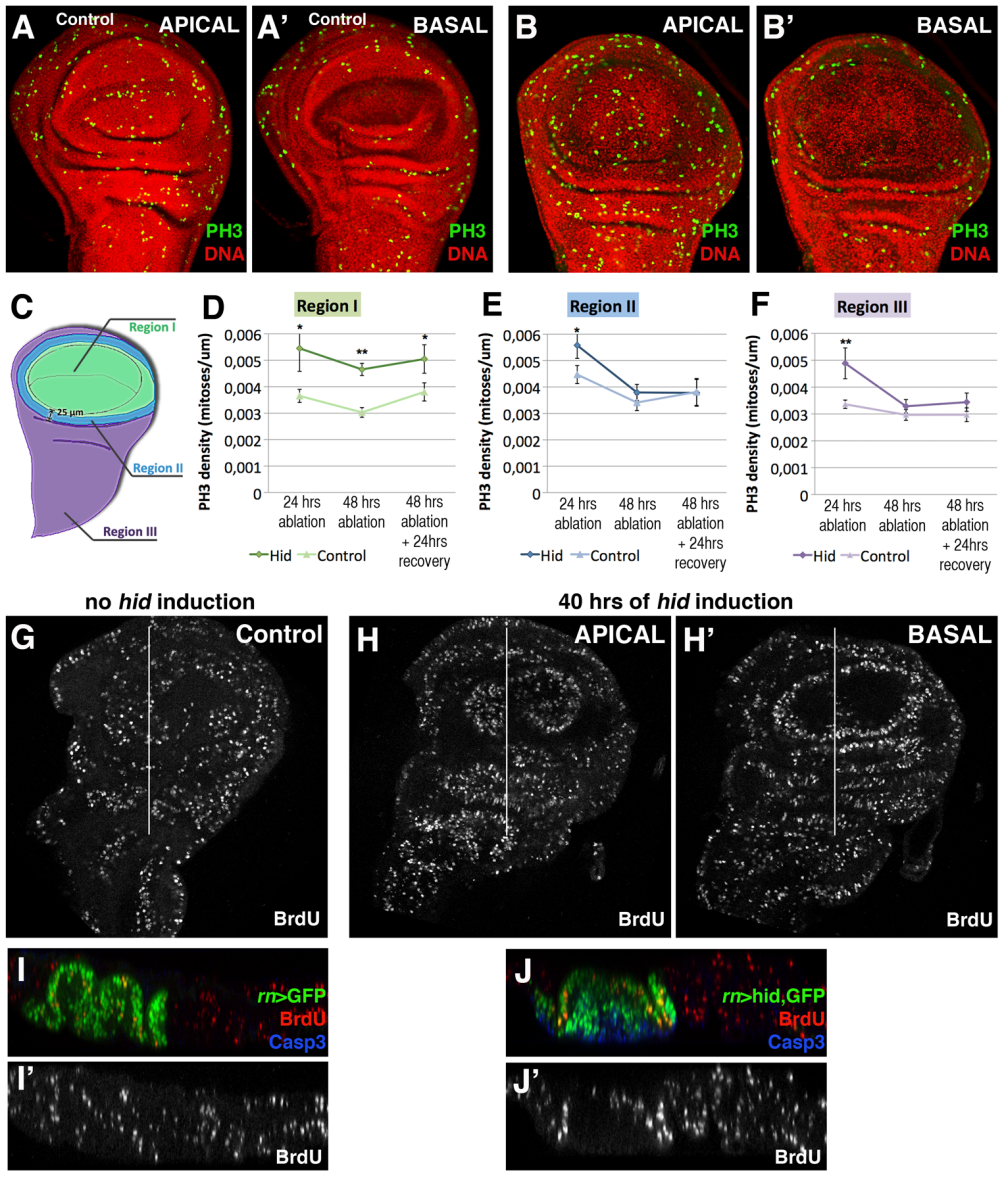
Proliferation pattern during and after *hid*-induced ablation of the Rn domain. (A–B′) PH3 (green) and nuclei (red) staining to show cells in M phase in a non-ablated disc (A–A′) and disc after 48 hrs of *hid* expression without recovery (B–B′). Apical and basal planes are shown for each disc. Notice the presence of numerous cells in mitosis in the apical plane of the disc under ablation indicating cells in the ablated domain are actively proliferating. In contrast, there are no mitotic cells in the basal region (B′) where the apoptotic debris accumulates. Note also that the density of cells in mitosis in the experimental disc (B) is higher than in control (A). To estimate the mitotic index in the disc under ablation we defined three regions, schematized in C): region I represents the Rn domain, region II comprises a ring of 25 µm away from region I, thus in contact with the damaged area, and region III covers the rest of the disc. Stacks of 20 focal planes of GFP or *hid*-expressing wing discs were obtained and the PH3 images were z-projected (excluding PH3 from adepithelial cells, which are attached to the notum region but have different origin and fate). The projected images, which contain the total proliferation rate of the disc, were segmented into the 3 regions and processed with Fiji software for automatic cell counting. The graphics show the mitotic index (density of PH3-labelled cells) in control (light colour) and experimental discs (dark colour) in regions I (D), II (E) and III (F) after 24 or 48 hrs of ablation and after allowing 24 hrs of recovery following the 48 hrs ablation time. In the controls these values were calculated after the corresponding times of GFP expression. Already 24 hrs after the beginning of the ablation there is a significant increase in the mitotic index in the three regions. Later in the ablation, this increase in the proliferation rate becomes restricted to region I. In all cases n = 15. Error bars represent SE (* = p<0,05; ** = p<0,1). (G–J′) BrdU incorporation marked cells in S phase in control (G) and (H, H′) wing discs after 40 hrs of *hid* expression. (I–J′) Transverse sections through the planes indicated by the white lines in G and H–H′. I–J panels show the merge and I′–J′ show the single channel of BrdU incorporation in control and discs under ablation, respectively.

The comparison of the mitotic index, calculated by the number of cells containing the phosphorylated form of Histone 3 (PH3) in *rn>hid* and in *rn>GFP* (control) discs at the end of a 24 hrs period at 29°C, indicates an increase of cells in mitosis in the *rn>hid* discs (Figure 3A–3B′). In addition, the examination of the distribution of mitotic cells reveals some significant features of the regeneration process. In the first place the density of cells in mitosis, though higher than in control, is not localised near the damaged tissue, but it is uniform over the disc, including the Rn domain (Figure 3B). We have calculated the mitotic index in three different regions of the control and the regenerating disc (Figure 3D–3F): region 1 covers the Rn domain, region 2 the area surrounding the domain and region 3 the rest of the disc. We find that already after 24 hrs of ablation the mitotic index is significantly higher (50%) in the disc under ablation than in the control, and that the increase is observed in the three regions (Figure 3D–3F). Altogether these data indicate that the localised cell killing elicits a systemic proliferative response that affects all regions of the disc. This response begins very soon after the damage has started. Interestingly, after 48 hrs the increase in the mitotic index appears restricted to region 1, corresponding to the damaged territory. This suggests that the initial systemic response is followed by a more localised one in the affected zone.

A second interesting feature is that the mitotic cells in the Rn domain under reconstruction localise in the apical section of the disc (Figure 3B) and are absent in the basal section (Figure 3B′), where the apoptotic debris accumulates. The presence of actively dividing cells in the domain at a time in which the pro-apoptotic Hid protein is still active provides additional support to the idea that the reconstruction process is concomitant with cell killing. It is very likely that many of the cells in division in the Rn domain are immigrant cells from outside.

The finding that there is an overall response to the localised damage is also supported by the results obtained examining BrdU incorporation, another indicator of actively dividing cells. We have compared BrdU levels in ablated and control discs (Figure 3G–3J′) after 40 hrs at 29°C. In both genotypes there is a homogenous incorporation in all regions of the discs, although the intensity of BrdU incorporation is higher in the ablated discs. The levels of BrdU incorporation after 24 and 48 hrs of Hid activity are similar inside and outside the reconstructed domain. We note the discrepancy between our results indicating a systemic response and those of Smith-Bolton et al 2009 and Bergantiños et al 2010, which suggest a local response. As discussed below, it suggests the possibility of different regenerative responses, perhaps depending on the kind of damage.

### Clonal analysis of discs with ablated domains

**Figure 4:**
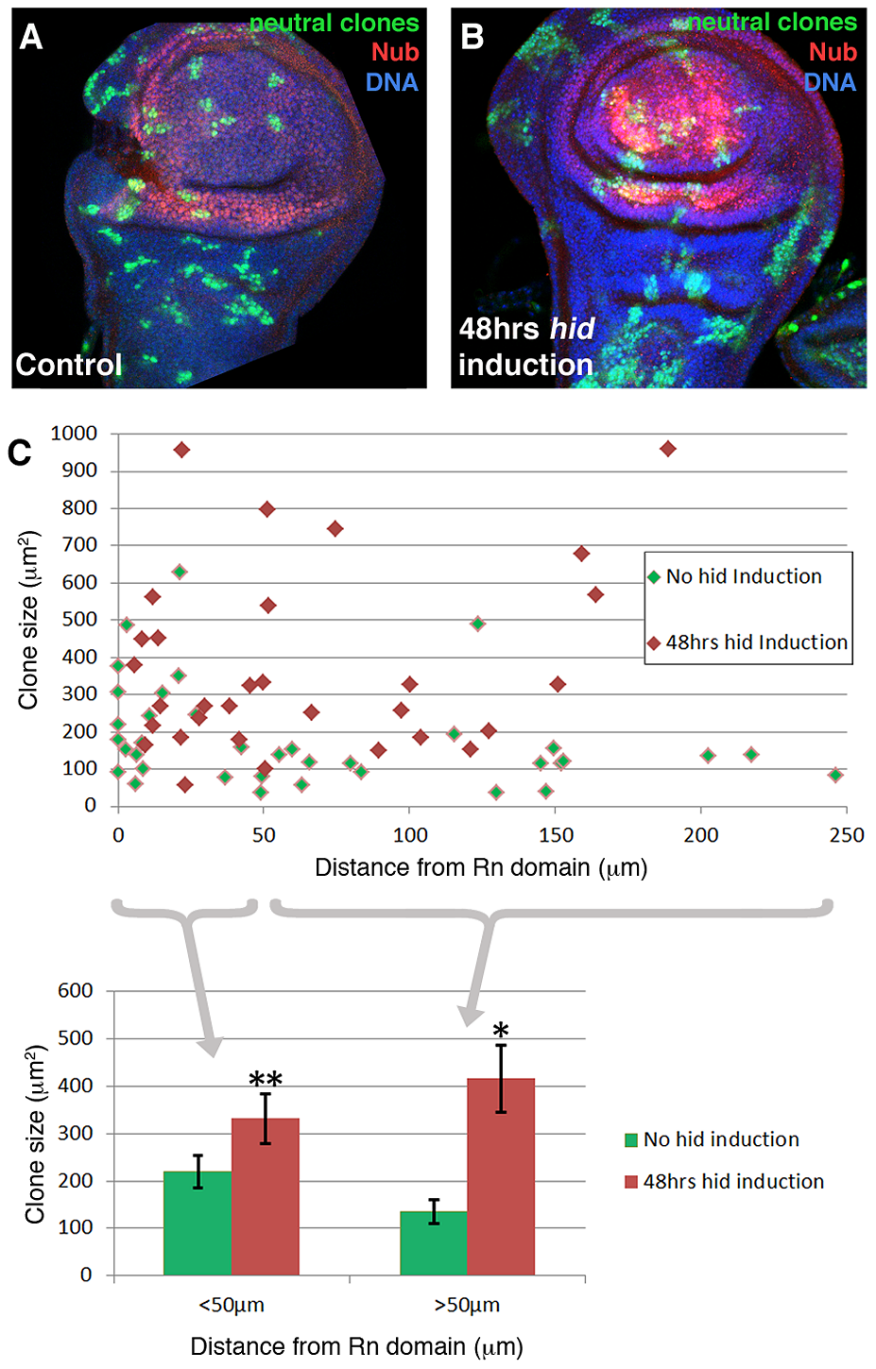
Clonal analysis of the wing disc under ablation. (A–B) Discs of genotype *hs-Flp; UAS-hid tub-Gal80^TS^/+; +/ubiP63E>stop>GFP* (control, A) and *hs-Flp; UAS-hid tub-Gal80^TS^/+; rn-Gal4/ubiP63E>stop>GFP* (experimental, B) containing neutral clones. The clones were induced at the beginning of the 48 hrs 29°C period and the discs fixed at the end. Thus in the experimental discs the clones assay the growth during the ablation period. Note the overall bigger size of the clones in the experimental discs and that the size appears to be homogenous over the disc. (C) Plot of the size of clones with respect to the distance to the border of the Nub domain; control clones in green, experimental in red. At the bottom of the figure, data from the plot discriminating clones located at <50 µm of distance and >50 µm. Note there is a homogeneous increase in the clone size that approximately doubles in the case of *hid* induction. This points to an additional round of replication in the whole disc. n>20 discs from 4 independent experiments. Error bars represent SE (* = p<0,05; ** = p = 0,07).

We have performed a clonal analysis of discs in which the Rn domain is under ablation. The genotype of the larvae is described in the Methods section. The clones were induced at the beginning of a 48 hrs ablation period and the discs fixed at the end. That is, the clones had been growing during the ablation time. Their size and shape was compared with control clones generated in larvae in which the temperature shift activated *UAS-GFP* instead of *UAS-hid*. We also considered the position of the clones in relation to the ablated domain. The results of this experiment are shown in Figure 4. The average size of the clones in the regenerating discs is about twice that of the controls. This is expected considering that the ablated Rn domain is about 40% of the disc total and the surviving cells have to perform additional divisions to approximately double the amount of tissue. In addition we find that the size of the clones is approximately the same in all the regions of the disc, including that of the clones inside the ablated domain. This can be visualised by plotting clone size with respect to the distance to the border of the Rn domain (Figure 4C). The average size of clones located closer that 50 µm to the Rn border is comparable to that of those located further than 50 µm. Thus the growth rate of the clones is similar in all regions of the disc. These observations strongly support the notion that there is a systemic response to the localised damage.

### Signalling response to the ablation

We have investigated the activity of the Dpp, Wg and JNK pathways in response to the ablation of the Rn and Sal^EPv^ domains. These pathways are known to be involved in growth control and response to damage in imaginal discs.

The Dpp pathway has a principal role as growth inducer in the wing disc; loss of Dpp activity inhibits cell division and reduces wing size, whereas high activity levels results in increased proliferation and in discs and wings of very large size [26], [27], [28]. Therefore we expected that the Dpp pathway would be involved in the growth response after injury.

**Figure 5:**
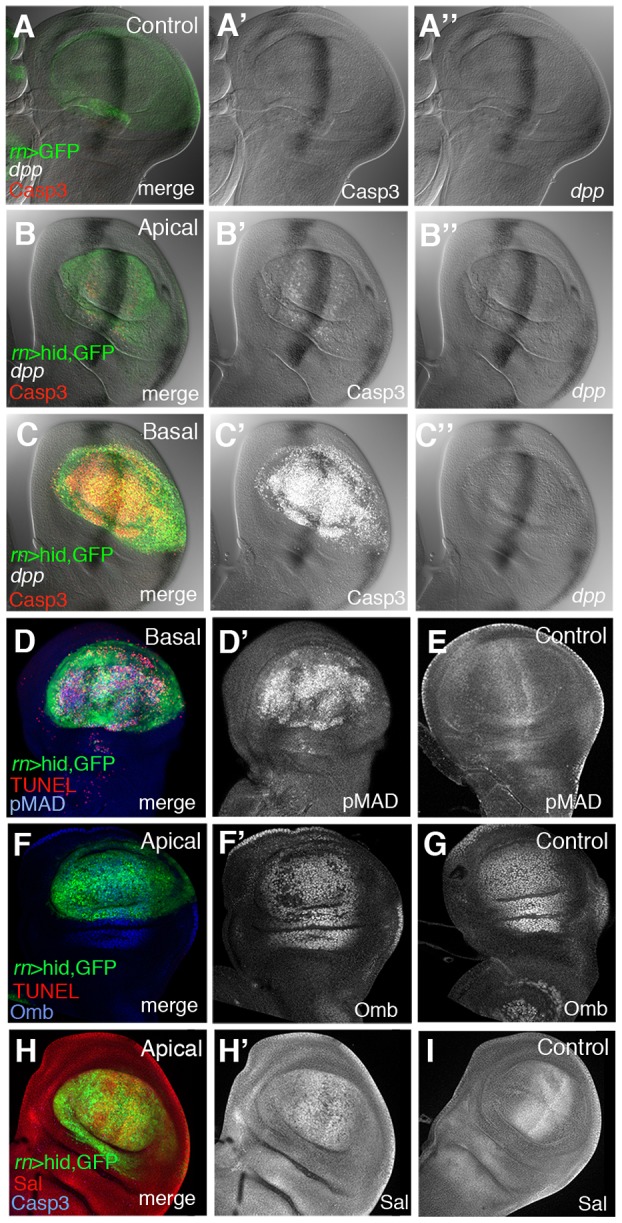
Dpp signalling in discs under ablation. (A–C″) Triple staining for *dpp* transcription and Caspase 3 and GFP activities in control *UAS-GFP/tub-Gal80^TS^; rn-Gal4* (A–A″) discs and in *UAS-hid tub-Gal80^TS^; rn-Gal4/*discs at the end of a 48 hrs period of ablation time. The B–B″ and C–C″ panels show the apical and basal regions of the experimental discs. Note that in the latter *dpp* transcription appears normal (B–C″) and similar to that in controls. Apoptotic cells, marked by the high levels of Caspase 3, accumulate in the basal region (C′) of the disc. (D–D′) Experimental disc stained for TUNEL and pMad activity at the end of the 48 hrs of ablation. The panel D shows the merge of channels in the basal plane of ablating disc. (D′) Elevated levels of pMad were associated with apoptotic cells at the basal plane of ablated disc. (E) pMAD expression in a control disc. (F–F′) Optomoter blind (Omb) expression at the end of the ablation period. Panel F show the merge of channels of triple staining (GFP, red for TUNEL and blue for Omb) in the apical plane of the disc. In the single channel (F′) Omb is shown in white for better visibility. Omb levels are practically normal in the apical plane (F′), where the expression is very similar to that of control discs (G). (H–H′) Spalt (Sal) expression at the end of the ablation period. Panel H shows the merge of channels in the apical planes of the disc under ablation. The levels of Sal (white) are close to normal in the apical plane of ablated disc (H′) and similar to that of a control disc (I).

We have first examined *dpp* expression during and after the ablation period. It was visualised by *in situ* hybridization with a specific probe and also using a *dpp*-*lacZ* insert. As shown in Figure 5 and Figure S5, after 20 or 40 hrs of ablation *dpp* expression remains normal; no significant alteration was found (Figure 5B″, 5C″), even though after 40 hrs of ablation there is a great deal of apoptosis in the Rn domain (Figure 5C′). We then examined the expression levels of the Dpp mediator *pMad* [4] and of two well known Dpp targets, *spalt* (*sal*) and *optomoter-blind (omb)*. The expression of *sal* and *omb* is essentially normal (Figure 5F′, 5H′) but, surprisingly, the activity of *pMad* appears uniformly elevated in the Rn domain (Figure 5D′).

Since *sal* and *omb* activities are mediated by *pMad* [4], it is hard to reconcile the normal expression of *sal* and *omb* seen in the damaged domain with the extended and elevated *pMad* expression. However, we find that the higher levels of *pMad* localise to the basal section of the disc, where the apoptotic cells accumulate (Figure 5D′). Also, double staining for TUNEL and pMad (Figure 5D) indicates that the nuclear fragments labelled with TUNEL contain the pMad protein. This suggests that the high levels of *pMad* observed in the experiments reflect a special feature of cells in apoptosis, but devoid of functional significance. We have explored the association of apoptosis and pMad accumulation in heavily irradiated discs, in which we observe high *pMad* levels associated with cells in apoptosis, but the expression of *dpp* remains normal (Figure S6).

Thus, considering all the evidence, our results indicate that the ablation of the Rn domain does not alter the activity of the Dpp pathway. It is worth pointing out that the normal Dpp activity is indeed required for the domain reconstruction; in experiments in which the *UAS-hid* transgene is replaced by *UAS-dpp-RNAi*, the elimination of *dpp* activity in the Rn domain for 48 hrs produces a phenotype similar to that of larvae that suffered continuous ablation (not shown).

**Figure 6:**
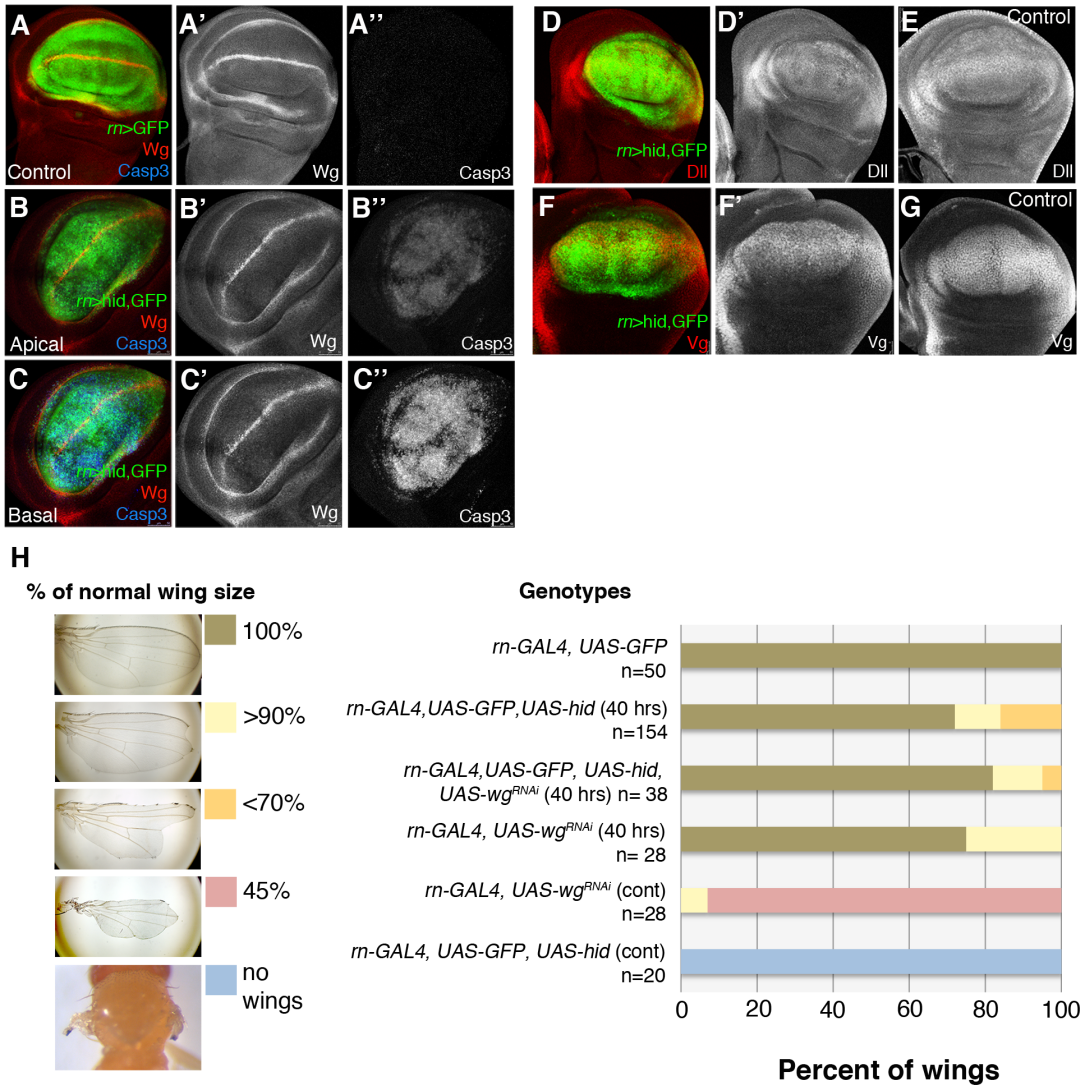
Wg signalling in discs under ablation. (A–A″) *wg* expression in a control *UAS-GFP/tub-Gal80^TS^; rn-Gal4* disc labelled for GFP, Wg (red) and Caspase 3 (blue). As in previous figures, single channels are in white for better visibility. (B–C″) The panels show Wg and Caspase 3 levels in an experimental *UAS-hid tub-Gal80^TS^; rn-Gal4* disc at the end of the ablation period. The expression of *wg* is not altered; cells in apoptosis accumulate in the basal section. (D–E) After 40 hrs of *hid*-induced ablation, *Distalless* (*Dll*) expression was similar in ablated (D′) and control (E) discs. (D) Merge of channels in ablated disc. (F–G) Similarly, vestigial (*Vg*) expression was normal in ablated discs (F′) and control discs (G). (F) Merge of channels in ablated disc. (H) Adult wing recovery after inhibition of Wg activity. Ablation induced by *rn GAL4 UAS hid* in larvae resulted in a range of wing sizes from normal to complete absence of wings (left panel), dependent on the genotype of the animal and the time of ablation (40 hrs or continuous ablation). The obtained data are represented in the graph and showed that depletion of Wg activity in the rotund domain during the time of ablation did not significantly change the percent of wings that recover the normal size after 40 hrs of *hid*-induced ablation.

Regarding the Wg pathway, it has been reported [14] that massive damage caused by over-expressing the TNF ligand Eiger [29], [30] causes strong *wg* up-regulation in the damaged tissue. However, in our experiments inducing apoptosis with Hid we do not find a significant alteration of *wg* expression. As shown in Figure 6, after 40 hrs of Hid activity *wg* expression appears normal, both inside and outside the ablated domain. Two target genes of the Wg pathway, *Distalless (Dll)* and *vestigial (vg)* [8] are also expressed normally (Figure 6D–6G). Experiments of ablation of the Sal^EPv^ domain yield similar results (Figure S4). Since there was the possibility of a function of the Wg pathway not easily detectable with the current labelling methods, we assayed the ability to regenerate of the Rn domain in conditions in which *wg* activity is inhibited, using a *UAS-wg-RNAi* line. We first showed that the *UAS-wg-RNAi* line effectively suppresses *wg* function (Figure 6H). The result, shown in Figure 6H, is that the Rn domain regenerates in the absence of *wg* function.

Thus, our results indicate that there is no significant response of the Wg pathway to the damage caused by Hid-dependent apoptosis. This result is in contrast with that reported by Smith-Bolton et al., 2009. In their experiments they used similar Gal4 drivers but different pro-apoptotic vectors, the TNF ligand Eiger (UAS-egr) and the pro-apoptotic gene *rpr*. We repeated their experiment in order to ascertain the experimental difference. We find that overexpression of Eiger and of Rpr indeed induce *wg* activity, although not too extensive in the case of Rpr (Figure S7). After 40 hrs of Eiger or Rpr activity the discs exhibit a distorted morphology (Figures S2 and S7), and do not recover a normal morphology even 48 hrs after the end of Eiger activity. This contrasts with our experiments using Hid, in which we observe full recovery after massive apoptosis. We believe that, especially Eiger overexpression, may cause other effects in addition to apoptosis and these effects interfere with the normal repair process.

**Figure 7:**
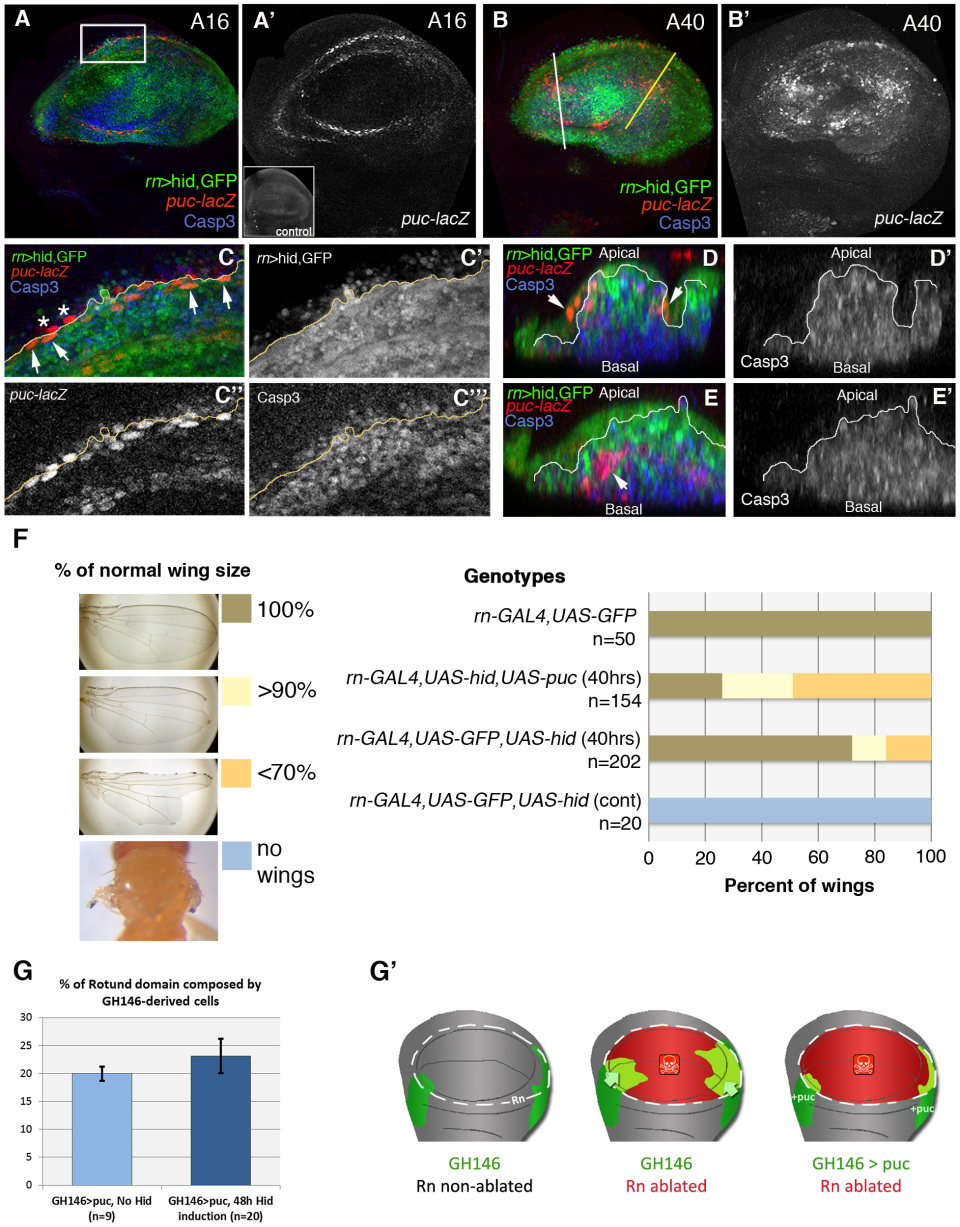
Activity of the JNK pathway during and after *hid*-induced ablation. (A–E′) After 16 hrs of *hid*-induced ablation, there is adventitious expression of the JNK target *puckered* (*puc*, labelled red or white), localized at the edges of the wound (A, A′), whereas after 40 hrs *puc-lacZ* expressing cells appear inside the damaged domain (B, B′). As shown in the inset in A′ there is no *puc* expression in the wing pouch of control discs. (C–C′″) Magnification of the area indicated in A. Merged (C) and separate channels of GFP (C′), *puc-lacZ* (C″) and Caspase-3 (C″) expression. Some of the *puc-lacZ* cells were localized inside the Rn domain and expressed GFP (arrows). However, a few of them were placed outside the rotund domain and did not express GFP (asterisks). The majority of *puc-lacZ* cells do not show Caspase3 activity. (D–E′) Transverse sections through the planes indicated by the white (D, D′) and yellow (E, E′) lines in B. Merge (D, E) and single channel of Caspase-3 activity (D′, E′). *puc-lacZ* cells were located at the apical plane of the wing disc and did not show Caspase-3 activity (arrows) (D). In other regions of the wing pouch, *puc-lacZ* cells showed Caspase-3 activity (arrows) and were extruded basally (E). (F) Adult wing recovery after inhibition of JNK activity in dying cells. The range of sizes is indicated in F, with the colour code. When JNK activity is specifically blocked in the Rn domain by overexpressing puc with the *UAS-puc* transgene, the percent of wings that recover the normal size after 40 hrs of *hid*-induced ablation decreased from 68% in controls to 25%. (G) Percentage of Rn domain derived from GH146 lineage after *puc*-overexpression in non-ablating conditions (light blue), and after 48 hrs of *hid* expression on Rn domain (dark blue). Compare with Figure 2I and note that while in the later there was an increase in the contribution of GH146 cells after ablation, here there is no significant difference. This suggests that inhibition of JNK prevents the migration of GH146 cells to the Rn domain. (G′) Diagrams schematizing the contribution of GH146 cell lineage to the Rn domain under different conditions: non-ablated discs (left); after the ablation of Rn domain, when the lineage increases its contribution (middle, see also Figure 2G–2H); and after Rn domain ablation with GH146 JNK-deficient cells (right).

Finally, we analysed the response of the JNK pathway to Hid-induced apoptosis. To monitor JNK activity we have used *a LacZ* insert in the *puckered (puc)* gene, a target of the pathway [31]. Normally the JNK pathway is not active in the Rn domain (Figure 7), but in the domain under reconstruction we observe many cells expressing the *puc-LacZ* transgene. These cells can be detected already 16 hrs after the commencement of the ablation and accumulate preferentially in the apical section of the disc (Figure 7), where the healthy cells are. We reasoned that these cells could be involved in the repair process. Besides, Bergantiños et al., 2010 have provided evidence that the JNK pathway is required for the regeneration of the patch domain. We tested the functional role of this adventitious JNK activity by inducing Hid activity in the Rn domain and at the same time inhibiting JNK by overexpressing the negative regulator *puc* [31]. By itself the overexpression of *puc* does not have any effect on the development of the disc (not shown). The degree of rescue was studied in adult wings, comparing the wings in which JNK can be up regulated with those in which the elevated levels of *puc* suppress JNK activity. The result is that the rescue of the Rn domain is significantly diminished (Figure 7F), supporting a role of the JNK pathway in the repair process.

We observed that some of the *puc*-expressing cells were not labelled with GFP, suggesting they come from outside the Rn domain (Figure 7C–7E′). Therefore we checked the effect of JNK in the immigration of cells towards the domain under repair. As mentioned above, the QF line GH146 is expressed preferentially in cells outside the Rn domain (Figure 2F) and the contribution of cells derived from the GH146 domain is increased significantly after massive damage in the Rn domain (Figure 2G, 2H). We have inhibited JNK activity in the GH146 cells by overexpressing *puc*. The result is that the contribution of these cells to the repopulation of the damaged Rn domain is now not increased after damage (Figure 7G). This experiment clearly suggests a requirement for JNK pathway activity in the immigration process.

## Discussion

We have utilised the pro-apoptotic gene *hid* to produce massive damage in the Rotund and Spalt domains in growing wing discs. The primary aim of these experiments was to study the response of the discs to the damage and their ability to regenerate the ablated domains.

It should be mentioned that at the time the damage is produced the discs are approximately at the middle of the larval period, at the beginning of the third stage. As the final number of cells in the mature disc is 31000 [5], 12400 (40%) would correspond to the Rn domain. We have estimated that during the third larval period the disc cells perform 3.5 cell divisions [5], therefore the at the time the ablation starts the whole disc should contain 2600 and the Rn domain about 1000 cells. At this stage the cells still have a high degree of developmental plasticity. For example, the developmental genes *wg* or *vestigial* (*vg*) are in early expression stages, quite different from the final expression patterns [32], [33].

Thus our experiments assay the regenerative response to massive damage of a growing blastema that is in a relatively early developmental stage. Another feature in our experiments is that the two domains under study are not restricted by lineage. That is, cells from outside the targeted domain share the lineage with those inside; in principle there should be no limitation to cells from outside contributing to reconstruct the damaged region.

There are three major findings that derive from our experiments:

### Massive cell death in disc domains provokes an immediate response aimed to restore the integrity of the disc

This reveals the existence of a powerful mechanism of tissue homeostasis that can cope with major lesions during development. Very shortly after the beginning of massive apoptosis in the Rn domain it is possible to observe immigrant cells (Figure 2) –identified by the marking method and in the lineage experiment using the QF system- that are occupying the place of the dying ones, which have been extruded to the basal section of the disc. The incorporation of these cells ensures the integrity of the epithelium and also that the domain develops normally, even during the ablation process. This is clearly manifest by examining the morphology of the disc and the pattern of expression of key developmental genes such *dpp, wg*, *vg, Dll, nub, rn, omb, sal* in the domain after 48 hrs of Hid activity and without allowing any time for recovery (Figure 1, Figure 5, Figure 6). All these genes are expressed normally, indicating that the immigrant cells have acquired the appropriate identity corresponding to the normal Rn domain. This is a striking result, as it demonstrates that the reconstruction process has occurred concomitantly with the massive cell killing. Previous studies [14], [15] have failed to observe this phenomenon, likely because those authors did not study the events during the ablation time; their analysis of the regeneration process was focused on the post-ablation period.

### The proliferative response necessary to restore the ablated domain is largely systemic

This result was also unexpected in view of published reports [14], [15] indicating that the ablation produces a local proliferative response around the damaged region. Our results, on the contrary, suggest that the response to the ablation of the Rn domain is largely systemic, as the increase in cell proliferation is generalised in the disc. The experimental support is as follows: 1) proliferation markers such as BrdU incorporation and PH3 staining are homogenously distributed over the regenerating disc (Figure 3). Also, accurate accounting of the number of mitotic (PH3 labelled) cells in three distinct regions reveals a significant increase of mitotic cells during ablation that affects the three regions (Figure 3), 2) Clonal analysis of the disc under ablation shows that while the average clone size is greater than in controls, the size increase is not localised, but general in the disc (Figure 4). However, the evolution of the mitotic index during and after the ablation (Figure 3) suggests that the initial systemic response in cell proliferation is followed by a local increase in the damaged zone.

We believe that these results can be interpreted in the following manner. There is a mechanism of overall control that continuously monitors compartment size during development and that blocks growth once the compartment has reached the final stereotyped size (see [34]). A massive damage like the ablation of the entire Rn domain is interpreted as a substantial diminution in size. This triggers a growth response in both the anterior and posterior compartments (the Rn domain extends to both), which is aimed to achieve normal size. Since the mechanism in question measures the overall size, the response is also general, increasing cell proliferation in all regions of the disc, including the domain under restoration.

We are aware that data from previous reports 14,15 suggest that the proliferative response is localised close to the damaged region. One possible reason for the difference is that the strong systemic response occurs during the first 24 hrs of ablation time (Figure 3), and is later followed by a more localised effect. Smith-Bolton et al 2009 restricted their analysis to post-ablation time, whereas Bergantiños et al 2010, only analysed the proliferative response in part of the disc.

### The Dpp and Wg signalling pathways do not play specific roles in the regenerative response, but the JNK pathway is specifically required

We do not see major alterations in the Dpp and Wg signalling pathways. The expression of *dpp* and *wg* remain essentially unaffected during and after ablation (Figure 5 and Figure 6). Moreover, their target genes *sal, omb, vg* and *Dll* are also expressed normally after 40 hrs of ablation. Intriguingly, we find elevated levels of pMad in the ablated tissue, but we believe that these high *pMad* levels are of little functional significance. One reason is that up regulation of *pMad* would be expected to induce a raise in the expression *sal* and *omb*, which is not observed (Figure 5). Besides, this accumulation of pMad appears associated only with cells in apoptosis, suggesting that it may be a peculiar property of apoptotic cells devoid of functional consequence. We wish to emphasise however that the reconstruction of the ablated domain does require the regular activity of the Dpp pathway, because in its absence there is no recovery of the ablated domain. This simply reflects the normal requirement of Dpp activity for the development of the wing structures.

The results obtained on *wg* deserve special attention. This gene has been implicated in the regeneration of the imaginal discs after transplantation [10] and there is evidence that Wnt genes are involved in regeneration in Hydra and in other organisms [12], [35]. Moreover, in experiments similar to ours, Smith-Bolton et al 2009 have shown that *wg* is up regulated, an observation that we have confirmed (Figure S7). Yet in the *rn>hid* regeneration experiments its expression is not altered and its function is not required (Figure 6). These observations raise the possibility of different regenerative responses depending on the kind and perhaps the developmental status of the damaged tissue.

Contrasting with the lack of response of the Dpp and Wg pathways, the activity of the JNK pathway is activated after damage and its function is necessary for the repair process (Figure 7), confirming prior work by Bergantiños et al (2010). In addition, our experiments inhibiting JNK in the cells close to the Rn domain (Figure 7G) strongly suggest that its activity is necessary to repopulate the domain under ablation, possibly facilitating the migration of cells. It is known that JNK activity plays a role in developmental processes like the embryonic dorsal closure or the sealing of the left and right sides of the discs [36], [37], that require cell movements, thus it may perform a similar function during the reconstruction of the damaged Rn or sal domains.

## Materials and Methods

### *Drosophila* strains and crosses

The *Drosophila* stocks used were *rn-Gal4* [19]; *sal^EPv^-Gal4* (gift of JF de Celis, CBMSO, Madrid, Spain); *tub-Gal80^ts^* [38]; *puc-lacZ* line (*puc^E69^*) and *UAS-puc^14C^* line [31]; *UAS-GFP* (BDSC), *UAS-shmi Dpp2* [39]; *UAS-wg^RNAi^* (VDRC 13352); *hs-Flp112* [40];QH146-QF line, QUAS-*Flp* line and QUAS-*GFP* line [23]; UAS-*Flp* (BDSC); *act*>stop>*lacZ* (gift of G Struhl); *ubi*>stop>GFP [41]; *UAS-Diap1^RNAi^* [42]; *UAS-eiger* [30]; *UAS-dp53.EX, UAS-hep^CA^, UAS-hid, UAS-rpr* and *P{dpp-lacZ.B}* lines are described in Flybase.

In the experiments using the Gal4/UAS system the general rule was to use only two UAS transgenes. This was to avoid the possibility of titrating the amount of Gal4 protein available for the UAS vectors.

### Clonal analysis

To induce neutral clones in the domain under ablation we heat shocked (12 min 37°C) larvae of genotype *hs-Flp; UAS-hid tub-Gal80^TS^/+; rn-Gal4/ubiP63E>stop>GFP* at the beginning of the 48 hrs ablation period. The discs were fixed immediately after the end of the Hid treatment. As controls we induced clones in *hs-Flp; UAS-hid tub-Gal80^TS^/+; ubiP63E>stop>GFP/+* larvae in which the temperature shift did not activate Hid.

### Dual Gal4/Q system experiments

The Q system is a binary expression system that is independent of the Gal4/UAS system [23]. In our experiments, we used the Gal4/UAS system to ablate the Rn domain, whereas with the GH146-QF line we traced the lineage of a group of cells preferentially located in the hinge area. The genotypes used were *UAS-hid tub-Gal80^TS^/QUAS-Flp ubi>stop>GFP ; +/GH146-QF* (control) and *UAS-hid tub-Gal80^TS^/QUAS-Flp ubi>stop>GFP ; rn-Gal4/GH146-QF* (*hid* induction in Rn domain

In the experiment to overexpress *puckered (puc)* the genotypes were *UAS-hid tub-Gal80^TS^/QUAS-puc QUAS-Flp ubi>stop>GFP ; +/GH146-QF* (control) and *UAS-hid tub-Gal80^TS^/QUAS-puc QUAS-Flp ubi>stop>GFP ; rn-Gal4/GH146-QF* (experimental)

### Construction of the QUAS-*puc* transgenic line

The *puckered* cDNA was obtained from the UAS-*puc* ^2A^ line [31] by PCR amplification and cloned into the pQUAST vector (Addgene plasmid 24349) as described in Potter et al., 2010. To enhance the construct expression and avoid positional effects of the integration, the BamH1 fragment from the resulting vector was cloned into pCa4B2G [43] at the BamH1 site. This vector flanks the insert with gypsy insulators and allows PhiC31-mediated integration [44]. The construction was integrated at the 51D locus.

### Immunostaining and *in situ* hybridization

Immunostaining and double antibody staining/*in situ* hybridization was performed as described previously [45]. Images were captured with a Leica (Solms, Germany) DB5500 B confocal microscope or a LSM510 Vertical (Zeiss, Thornwood, NY, USA) confocal microscope.

The following primary antibodies were used: rabbit anti-Caspase 3 (Roche) 1∶50; mouse anti-ß-Galactosidase (DSHB 40-1a) 1∶50; mouse anti-BrdU (Roche) 1∶10; rabbit anti-Vestigial [46] 1∶500; mouse anti-Distalless [47] 1∶400; rabbit anti-Omb (gift of G. O. Pflugfelder) 1∶100; rat anti-Spalt (gift of JF de Celis, CBMSO, Madrid, Spain) 1∶50; rabbit anti-pMAD [48] 1∶100; mouse anti-Wingless (DSHB 4D4) 1∶50; anti-Digoxigenin-AP (Roche, Basel, Switzerland) 1∶4000; anti-Phospho-Histone H3 (Millipore); rabbit anti-Rotund 1∶250 and rabbit anti-Nubbin 1∶250(gifts of F.Diaz-Benjumea, CBMSO, Madrid, Spain)

Fluorescently labelled secondary antibodies (Molecular Probes Alexa) were used in a 1∶200 dilution. TO-PRO3 (Invitrogen) was used in a 1∶600 dilution to label nuclei; Phalloidin-TRITC (Sigma) and Phalloidin-Cy5 were used in a 1∶200 dilution to label the F-actin network.

The RNA probe for the *dpp* transcript was obtained using the BDGP cDNA clone RE20611 as a template.

### Histochemical detection of ß-Galactosidase activity and BrdU incorporation

Activity of the *lacZ* gene was visualised by staining for ß-gal activity, as described in [49].

For Bromo-2′-deoxyuridine (BrdU) incorporation, wing imaginal discs were cultured in PBS 1x medium supplemented with 1 mg/mL BrdU (Roche) for 30 minutes at room temperature. Discs were subsequently washed in PBS 1x, fixed in 4% Paraformaldehyde, 0.1% Triton, 0.1% DOC for 1 h at room temperature, treated with RQ1 DNase (Promega) for 2 hours and fixed again in 4% Paraformaldehyde, 0.1% Triton, 0.1% DOC for 30 minutes. Discs were then immunostained as described above.

### TUNEL assay

Apoptotic cells were detected based on labeling of DNA strand breaks using TUNEL technology (In situ Cell Death Detection kit, TMR red, Roche).

### Slide-mounting adult wings

Adult wings were mounted in Euparal Mounting medium after having dissected the flies in a mixture of alcohol/glycerine. Images were captured with a Leica microscope

## Supporting Information

Figure S1 Ablation of the Rn and Sal domains by the Gal4/UAS/Gal80TS method. The Rotund (A) and the Spalt (B) domains in the wing imaginal disc as defined in wing discs of genotype *rn-Gal4>UAS-GFP and sal^EPv^-Gal4-UAS-GFP*. (C) Control adult wing. (D, E) Adult wing phenotypes obtained after allowing the continuous activity of Hid in the Rotund (D) or Spalt (E) domains. (F) Standard protocol used to study ablation and regeneration: animals were raised at 17°C until day 7 after egg-laying and shifted to 29°C for various lengths of time. Larvae were then returned to 17°C to allow recovery or were dissected at the indicated time points. (G) Levels of recovery of the wing after 24, 48 and 72 hrs of *hid*-induced ablation at 29°C.
(TIF)

Figure S2 Alterations induced by different pro-apoptotic vectors in the Rotund domain. Confocal images of wing discs of different combinations of the rn-Gal4 driver with various pro-apoptotic transgenes. We have examined the levels of apoptosis in the Rn domain and the overall disc morphology after 24 and 48 hrs of expression of *UAS-hid* (A–A″), *UAS-p53* (B–B″), *UAS-diap1^RNAi^* (C–C″), *UAS-hep^CA^* (D–D″), *UAS-egr* (E–E″) and *UAS-rpr* (F–F″). The images show transversal sections perpendicular to the A/P border and are oriented with the peripodial membrane at the top and the columnar epithelium at the bottom. For each pro-apoptotic factor the two top panels show the evolution of morphology of the Rn domain after 24 and 48 hours of induction. The lower panels show staining after 48 hours of induction with anti-Crumbs (green), used to mark the apical side of epithelial cells, and anti-Casp3 (red) to mark apoptosis. Note the high amount of apoptotic corpses after *hid* overexpression in (A). Note also the presence of ectopic folding and apoptotic debris trapped in the disc lumen in the cases of *hep* ^CA^ (D), *egr* (E) and *rpr* (F) expression. The morphology of the disc is abnormal, especially in the case of rn>egr disc. This may indicate the occurrence of additional effects caused by the activity of those factors. Panels G, H and I show the morphology of 48 hrs ablated discs with *hid*, *eiger* or *reaper*, and after 48 hrs of recovery. The discs are stained with the wing pouch marker Nubbin. Note the incomplete reconstruction of the Nubbin domain after ablation with *eiger* (H) or *reaper* (I), even after 48 hrs of recovery. Additional information in Table S1.
(TIF)

Figure S3 Method lo label all the cells of the Rn domain. Wing discs of genotype *rn-Gal4 tub-Gal80^TS^ UAS-GFP UAS-Flp act>stop>lacZ* (A–A″, control) and *rn-Gal4 tub-Gal80^TS^ UAS-hid UAS-Flp act>stop>lacZ* (B–B″, experimental) after 48 hrs of GFP (A–A″) or *hid* (B–B″) overexpression at 29°C. Note the lineage label in nearly 100% of *rn*-expressing cells (GFP-positive) in the control disc (A″) and the co-expression of apoptosis and lineage label in the ablated disc (B″).
(TIF)

Figure S4 Ablation and reconstruction of the Sal^EPv^ domain. (A–D) Orthogonal sections perpendicular to the A/P border (at the point of maximum width of the Sal^EPv^ domain) of *sal^EPv^-Gal4 UAS-GFP/tub-Gal80^TS^; UAS-Flp act>stop>LacZ* (control) (A–B) and *sal^EPv^-Gal4/UAS-hid tub-Gal80^TS^; UAS-Flp act>stop>LacZ* (C–D) genotypes. Inductions times are indicated at the left, and the outline of the living epithelium (excluding apoptotic cells) is marked with a white line. Asterisks point to apoptotic cells which are being extruded basally (also visible as nuclear fragments). In (D), note that there are cells positive for Sal staining (green), which do not belong to the former Sal domain as they express low βgal activity, indicating *de novo* acquisition of Sal identity driven by a late recombination of the cassette. (E–E″) *wg* expression in non-ablating disc. (F–F″) Wg levels in ablated disc after 40 hrs of *hid*-expression in the Sal^EPv^ domain. (E, F) Merge and separate channels of Wg (E′. F′) and Caspase-3 (E″, F″) expression. Note that expression of *wg* is not altered. (G–I′) *Dll* expression (red) in control (G–G′) and apical (H. H′) or basal (I, I′) planes of ablated disc at 40 hrs of *hid*-induced ablation in the Sal^EPv^ domain. (J–L′) *Vg* expression (red) in control (J, J′) and apical (K, K′) or basal (L, L′) planes of ablated disc after 40 hrs of *hid*-induced ablation. The expression of both *Dll* and *Vg* in the apical plane remain essentially normal.
(TIF)

Figure S5 *dpp* expression after *hid*-induced ablation. (A–A″) *dpp-lacZ* expression in a control disc. (B–B″) *dpp-lacZ* expression in a rn>hid wing disc after 20 hrs of *hid*-induced ablation. (C–C″) *dpp-lacZ* expression in a rn>hid disc after 40 hrs of *hid*-induced ablation. *dpp* expression is label in red, the Rn domain in green and caspase activity in blue. Note that *dpp* expression remains normal during ablation even though Casp3 activity is very high. (A, B, C) Merges and separate channels of *dpp-lacZ* expression (A′, B′, C′) and Caspase-3 levels (A″, B″, C″).
(TIF)

Figure S6 pMAD levels after massive irradiation. Third instar larvae were irradiated with 3000 rads and dissected 24 hrs after irradiation. Wing discs were extracted, fixed and immunostained as described in Experimental Procedures. (A–A″) pMAD expression at the A/P border in non-irradiated wing discs. (B–B″) pMAD expression localized in apoptotic cells (marked by TUNEL staining) in discs subjected to irradiation. (C–C″) Magnification of B–B″ images. Note the correspondence of TUNEL and pMad labels (D–D′″) Another disc showing massive apoptosis after irradiation. The high levels of pMAD correlate with TUNEL, but *dpp* expression remains normal.
(TIF)

Figure S7 *wg* up regulation after *eiger*- or *reaper*-induced ablation. As previously reported [14], we observed a strong Wg up regulation in regenerating discs, after *eiger* overexpression in the Rotund domain. (A–A″) *wg* expression in control disc. (B–B″) *wg* up regulation in an *rn>egr* disc after 40 hrs of *eiger* expression. (C–C″) *wg* up regulation in an *rn>rpr* disc after 40 hrs of *reaper* expression.
(TIF)

Table S1 Summary of the effects induced by different pro-apoptotic vectors in the Rotund domain, as shown in Figures S2 and S7. In the row reporting the contact between the Peripodial and Columnar epithelia we distinguish between normal contact and lack of contact, which results in the appearance of a blister in the lumen. The rest is self explanatory. n.a. not analysed.
(DOCX)

## References

[1] Morgan TH (1901) Regeneration and Liability to Injury. Science 14: 235–248. 17806597 10.1126/science.14.346.235

[2] Cohen SM (1993) Imaginal disc development. The Development of Drosophila melanogaster Cold Spring Harbor Laboratory Press Volume II: 747–841.

[3] Hadorn E (1978) Imaginal discs: transdetermination. Ashburner M, Wright TRF, editors The Genetics and Biology of Drosophila 2c Academic Press; New York: 555–617.

[4] Affolter M, Basler K (2007) The Decapentaplegic morphogen gradient: from pattern formation to growth regulation. Nat Rev Genet 8: 663–674. 17703237 10.1038/nrg2166

[5] Martin FA, Herrera SC, Morata G (2009) Cell competition, growth and size control in the Drosophila wing imaginal disc. Development 136: 3747–3756. 19855017 10.1242/dev.038406

[6] Bryant PJ (1975) Pattern formation in the imaginal wing disc of Drosophila melanogaster: fate map, regeneration and duplication. J Exp Zool 193: 49–77. 806653 10.1002/jez.1401930106

[7] Calleja M, Herranz H, Estella C, Casal J, Lawrence P, et al (2000) Generation of medial and lateral dorsal body domains by the pannier gene of Drosophila. Development 127: 3971–3980. 10952895 10.1242/dev.127.18.3971

[8] Tabata T, Takei Y (2004) Morphogens, their identification and regulation. Development 131: 703–712. 14757636 10.1242/dev.01043

[9] Haynie JL, Bryant PJ (1976) Intercalary regeneration in imaginal wing disk of Drosophila melanogaster. Nature 259: 659–662. 814470 10.1038/259659b0

[10] McClure KD, Sustar A, Schubiger G (2008) Three genes control the timing, the site and the size of blastema formation in Drosophila. Dev Biol 319: 68–77. 18485344 10.1016/j.ydbio.2008.04.004 PMC2483308

[11] Schubiger M, Sustar A, Schubiger G (2010) Regeneration and transdetermination: the role of wingless and its regulation. Dev Biol 347: 315–324. 20816798 10.1016/j.ydbio.2010.08.034 PMC2976676

[12] Chera S, Ghila L, Dobretz K, Wenger Y, Bauer C, et al (2009) Apoptotic cells provide an unexpected source of Wnt3 signaling to drive hydra head regeneration. Dev Cell 17: 279–289. 19686688 10.1016/j.devcel.2009.07.014

[13] Stoick-Cooper CL, Weidinger G, Riehle KJ, Hubbert C, Major MB, et al (2007) Distinct Wnt signaling pathways have opposing roles in appendage regeneration. Development 134: 479–489. 17185322 10.1242/dev.001123

[14] Smith-Bolton RK, Worley MI, Kanda H, Hariharan IK (2009) Regenerative growth in Drosophila imaginal discs is regulated by Wingless and Myc. Dev Cell 16: 797–809. 19531351 10.1016/j.devcel.2009.04.015 PMC2705171

[15] Bergantinos C, Corominas M, Serras F (2010) Cell death-induced regeneration in wing imaginal discs requires JNK signalling. Development 137: 1169–1179. 20215351 10.1242/dev.045559

[16] Sun G, Irvine KD (2011) Regulation of Hippo signaling by Jun kinase signaling during compensatory cell proliferation and regeneration, and in neoplastic tumors. Developmental biology 350: 139–151. 21145886 10.1016/j.ydbio.2010.11.036 PMC3038240

[17] Grusche FA, Degoutin JL, Richardson HE, Harvey KF (2011) The Salvador/Warts/Hippo pathway controls regenerative tissue growth in Drosophila melanogaster. Developmental biology 350: 255–266. 21111727 10.1016/j.ydbio.2010.11.020

[18] Perez-Garijo A, Shlevkov E, Morata G (2009) The role of Dpp and Wg in compensatory proliferation and in the formation of hyperplastic overgrowths caused by apoptotic cells in the Drosophila wing disc. Development 136: 1169–1177. 19244279 10.1242/dev.034017

[19] St Pierre SE, Galindo MI, Couso JP, Thor S (2002) Control of Drosophila imaginal disc development by rotund and roughened eye: differentially expressed transcripts of the same gene encoding functionally distinct zinc finger proteins. Development 129: 1273–1281. 11874922 10.1242/dev.129.5.1273

[20] Barrio R, de Celis JF (2004) Regulation of spalt expression in the Drosophila wing blade in response to the Decapentaplegic signaling pathway. Proc Natl Acad Sci U S A 101: 6021–6026. 15079076 10.1073/pnas.0401590101 PMC395916

[21] Agnel M, Roder L, Vola C, Griffin-Shea R (1992) A Drosophila rotund transcript expressed during spermatogenesis and imaginal disc morphogenesis encodes a protein which is similar to human Rac GTPase-activating (racGAP) proteins. Mol Cell Biol 12: 5111–5122. 1406685 10.1128/mcb.12.11.5111-5122.1992 PMC360445

[22] Ng M, Diaz-Benjumea FJ, Cohen SM (1995) Nubbin encodes a POU-domain protein required for proximal-distal patterning in the Drosophila wing. Development 121: 589–599. 7768195 10.1242/dev.121.2.589

[23] Potter CJ, Tasic B, Russler EV, Liang L, Luo L (2010) The Q system: a repressible binary system for transgene expression, lineage tracing, and mosaic analysis. Cell 141: 536–548. 20434990 10.1016/j.cell.2010.02.025 PMC2883883

[24] Abbott LC, Karpen GH, Schubiger G (1981) Compartmental restrictions and blastema formation during pattern regulation in Drosophila imaginal leg discs. Dev Biol 87: 64–75. 7286422 10.1016/0012-1606(81)90061-0

[25] O'Brochta DA, Bryant PJ (1987) Distribution of S-phase cells during the regeneration of Drosophila imaginal wing discs. Dev Biol 119: 137–142. 3098601 10.1016/0012-1606(87)90215-6

[26] Burke R, Basler K (1996) Dpp receptors are autonomously required for cell proliferation in the entire developing Drosophila wing. Development 122: 2261–2269. 8681806 10.1242/dev.122.7.2261

[27] Martin-Castellanos C, Edgar BA (2002) A characterization of the effects of Dpp signaling on cell growth and proliferation in the Drosophila wing. Development 129: 1003–1013. 11861483 10.1242/dev.129.4.1003

[28] Martin FA, Perez-Garijo A, Moreno E, Morata G (2004) The brinker gradient controls wing growth in Drosophila. Development 131: 4921–4930. 15371310 10.1242/dev.01385

[29] Igaki T, Kanda H, Yamamoto-Goto Y, Kanuka H, Kuranaga E, et al (2002) Eiger, a TNF superfamily ligand that triggers the Drosophila JNK pathway. EMBO J 21: 3009–3018. 12065414 10.1093/emboj/cdf306 PMC126061

[30] Moreno E, Yan M, Basler K (2002) Evolution of TNF signaling mechanisms: JNK-dependent apoptosis triggered by Eiger, the Drosophila homolog of the TNF superfamily. Curr Biol 12: 1263–1268. 12176339 10.1016/s0960-9822(02)00954-5

[31] Martin-Blanco E, Gampel A, Ring J, Virdee K, Kirov N, et al (1998) puckered encodes a phosphatase that mediates a feedback loop regulating JNK activity during dorsal closure in Drosophila. Genes Dev 12: 557–570. 9472024 10.1101/gad.12.4.557 PMC316530

[32] Kim J, Sebring A, Esch JJ, Kraus ME, Vorwerk K, et al (1996) Integration of positional signals and regulation of wing formation and identity by Drosophila vestigial gene. Nature 382: 133–138. 8700202 10.1038/382133a0

[33] Whitworth AJ, Russell S (2003) Temporally dynamic response to Wingless directs the sequential elaboration of the proximodistal axis of the Drosophila wing. Dev Biol 254: 277–288. 12591247 10.1016/s0012-1606(02)00036-2

[34] Morata G, Herrera SC (2010) Differential division rates and size control in the wing disc. Fly (Austin) 4. 10.4161/fly.4.3.11516 20224294

[35] Lin G, Slack JM (2008) Requirement for Wnt and FGF signaling in Xenopus tadpole tail regeneration. Developmental biology 316: 323–335. 18329638 10.1016/j.ydbio.2008.01.032

[36] Jacinto A, Woolner S, Martin P (2002) Dynamic analysis of dorsal closure in Drosophila: from genetics to cell biology. Dev Cell 3: 9–19. 12110163 10.1016/s1534-5807(02)00208-3

[37] Pastor-Pareja JC, Grawe F, Martin-Blanco E, Garcia-Bellido A (2004) Invasive cell behavior during Drosophila imaginal disc eversion is mediated by the JNK signaling cascade. Dev Cell 7: 387–399. 15363413 10.1016/j.devcel.2004.07.022

[38] McGuire SE, Le PT, Osborn AJ, Matsumoto K, Davis RL (2003) Spatiotemporal rescue of memory dysfunction in Drosophila. Science 302: 1765–1768. 14657498 10.1126/science.1089035

[39] Haley B, Hendrix D, Trang V, Levine M (2008) A simplified miRNA-based gene silencing method for Drosophila melanogaster. Dev Biol 321: 482–490. 18598689 10.1016/j.ydbio.2008.06.015 PMC2661819

[40] Struhl G, Basler K (1993) Organizing activity of wingless protein in Drosophila. Cell 72: 527–540. 8440019 10.1016/0092-8674(93)90072-x

[41] Evans CJ, Olson JM, Ngo KT, Kim E, Lee NE, et al (2009) G-TRACE: rapid Gal4-based cell lineage analysis in Drosophila. Nat Methods 6: 603–605. 19633663 10.1038/nmeth.1356 PMC2754220

[42] Leulier F, Ribeiro PS, Palmer E, Tenev T, Takahashi K, et al (2006) Systematic in vivo RNAi analysis of putative components of the Drosophila cell death machinery. Cell Death Differ 13: 1663–1674. 16485033 10.1038/sj.cdd.4401868

[43] Markstein M, Pitsouli C, Villalta C, Celniker SE, Perrimon N (2008) Exploiting position effects and the gypsy retrovirus insulator to engineer precisely expressed transgenes. Nat Genet 40: 476–483. 18311141 10.1038/ng.101 PMC2330261

[44] Groth AC, Fish M, Nusse R, Calos MP (2004) Construction of transgenic Drosophila by using the site-specific integrase from phage phiC31. Genetics 166: 1775–1782. 15126397 10.1534/genetics.166.4.1775 PMC1470814

[45] Shlevkov E, Morata G (2012) A dp53/JNK-dependant feedback amplification loop is essential for the apoptotic response to stress in Drosophila. Cell Death Differ 19: 451–460. 21886179 10.1038/cdd.2011.113 PMC3278728

[46] Williams JA, Bell JB, Carroll SB (1991) Control of Drosophila wing and haltere development by the nuclear vestigial gene product. Genes Dev 5: 2481–2495. 1752439 10.1101/gad.5.12b.2481

[47] Duncan DM, Burgess EA, Duncan I (1998) Control of distal antennal identity and tarsal development in Drosophila by spineless-aristapedia, a homolog of the mammalian dioxin receptor. Genes Dev 12: 1290–1303. 9573046 10.1101/gad.12.9.1290 PMC316766

[48] Martin FA, Morata G (2006) Compartments and the control of growth in the Drosophila wing imaginal disc. Development 133: 4421–4426. 17035294 10.1242/dev.02618

[49] Busturia A, Morata G (1988) Ectopic expression of homeotic genes caused by the elimination of the Polycomb gene in Drosophila imaginal epidermis. Development 104: 713–720. 2908325 10.1242/dev.104.4.713

