# Supplementary figures and images for: Tissue Homeostasis in the Wing Disc of Drosophila melanogaster: Immediate Response to Massive Damage during Development
Source: PLoS Genet. 2013 Apr 25;9(4):e1003446. doi: 10.1371/journal.pgen.1003446 (PMC3636033; doi:10.1371/journal.pgen.1003446)

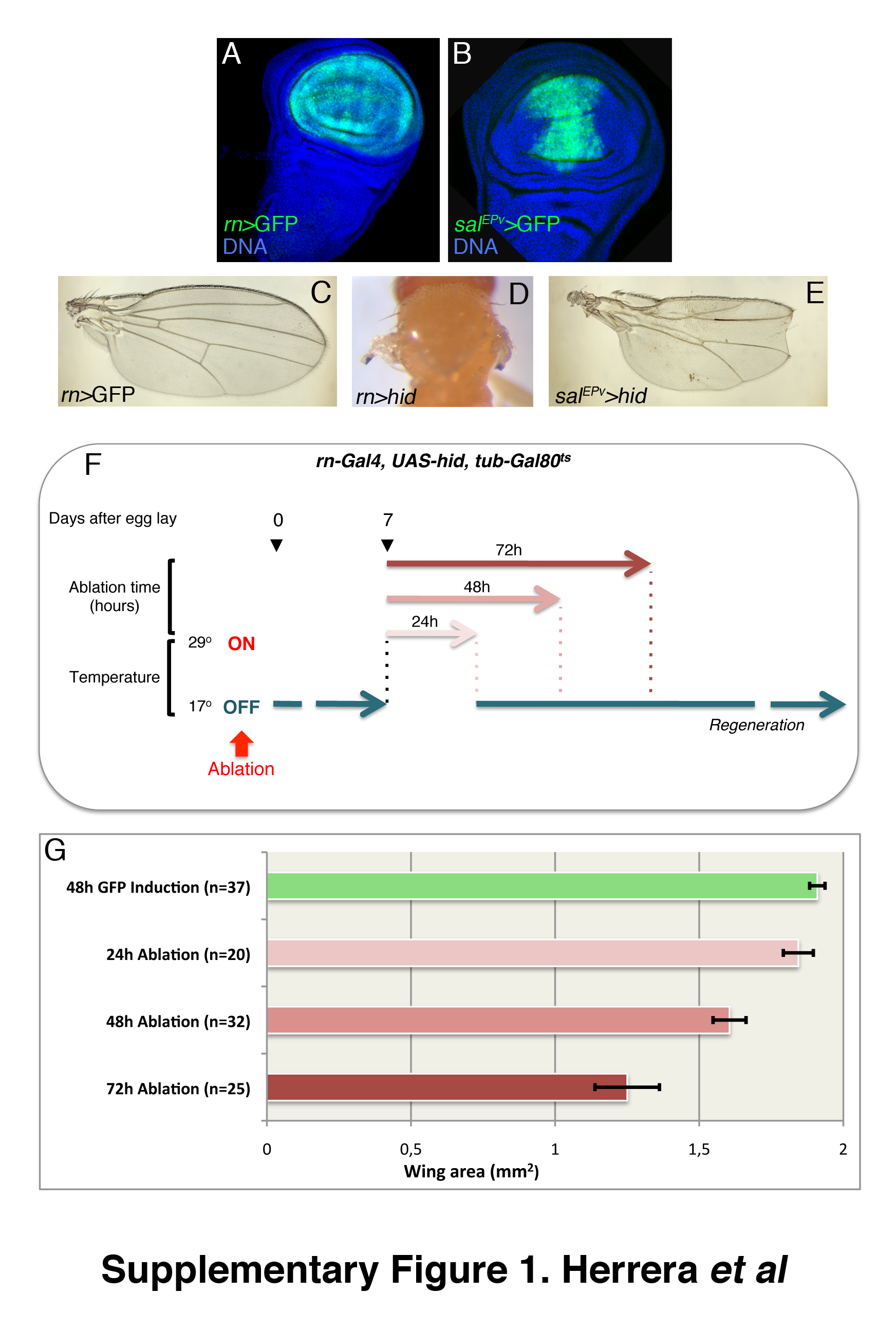

Supplement: Figure S1 — Ablation of the Rn and Sal domains by the Gal4/UAS/Gal80TS method. The Rotund (A) and the Spalt (B) domains in the wing imaginal disc as defined in wing discs of genotype rn-Gal4>UAS-GFP and salEPv-Gal4-UAS-GFP. (C) Control adult wing. (D, E) Adult wing phenotypes obtained after allowing the continuous activity of Hid in the Rotund (D) or Spalt (E) domains. (F) Standard protocol used to study ablation and regeneration: animals were raised at 17°C until day 7 after egg-laying and shifted to 29°C for various lengths of time. Larvae were then returned to 17°C to allow recovery or were dissected at the indicated time points. (G) Levels of recovery of the wing after 24, 48 and 72 hrs of hid-induced ablation at 29°C. (TIF) [file pgen.1003446.s001.tif]

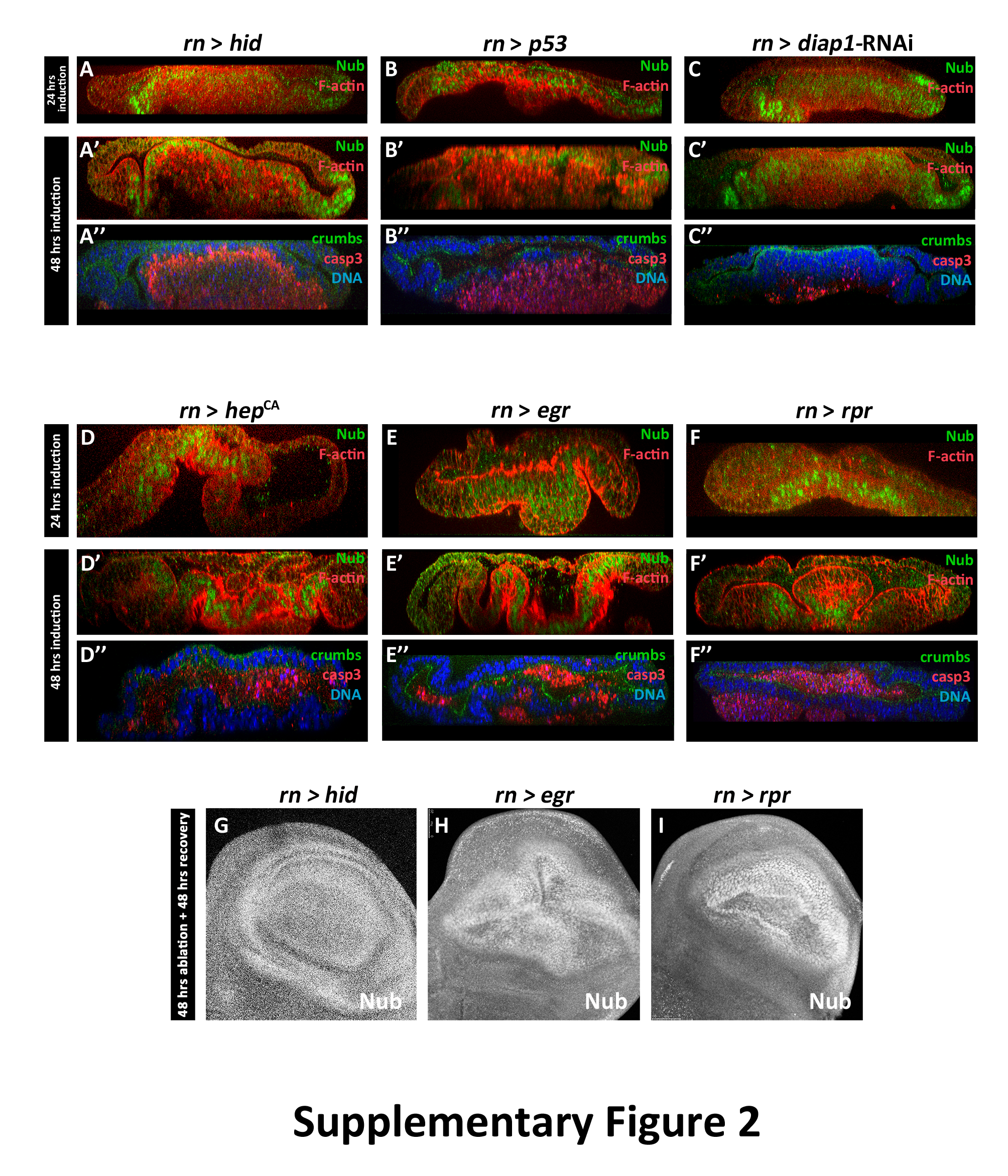

Supplement: Figure S2 — Alterations induced by different pro-apoptotic vectors in the Rotund domain. Confocal images of wing discs of different combinations of the rn-Gal4 driver with various pro-apoptotic transgenes. We have examined the levels of apoptosis in the Rn domain and the overall disc morphology after 24 and 48 hrs of expression of UAS-hid (A–A″), UAS-p53 (B–B″), UAS-diap1RNAi (C–C″), UAS-hepCA (D–D″), UAS-egr (E–E″) and UAS-rpr (F–F″). The images show transversal sections perpendicular to the A/P border and are oriented with the peripodial membrane at the top and the columnar epithelium at the bottom. For each pro-apoptotic factor the two top panels show the evolution of morphology of the Rn domain after 24 and 48 hours of induction. The lower panels show staining after 48 hours of induction with anti-Crumbs (green), used to mark the apical side of epithelial cells, and anti-Casp3 (red) to mark apoptosis. Note the high amount of apoptotic corpses after hid overexpression in (A). Note also the presence of ectopic folding and apoptotic debris trapped in the disc lumen in the cases of hep CA (D), egr (E) and rpr (F) expression. The morphology of the disc is abnormal, especially in the case of rn>egr disc. This may indicate the occurrence of additional effects caused by the activity of those factors. Panels G, H and I show the morphology of 48 hrs ablated discs with hid, eiger or reaper, and after 48 hrs of recovery. The discs are stained with the wing pouch marker Nubbin. Note the incomplete reconstruction of the Nubbin domain after ablation with eiger (H) or reaper (I), even after 48 hrs of recovery. Additional information in Table S1. (TIF) [file pgen.1003446.s002.tif]

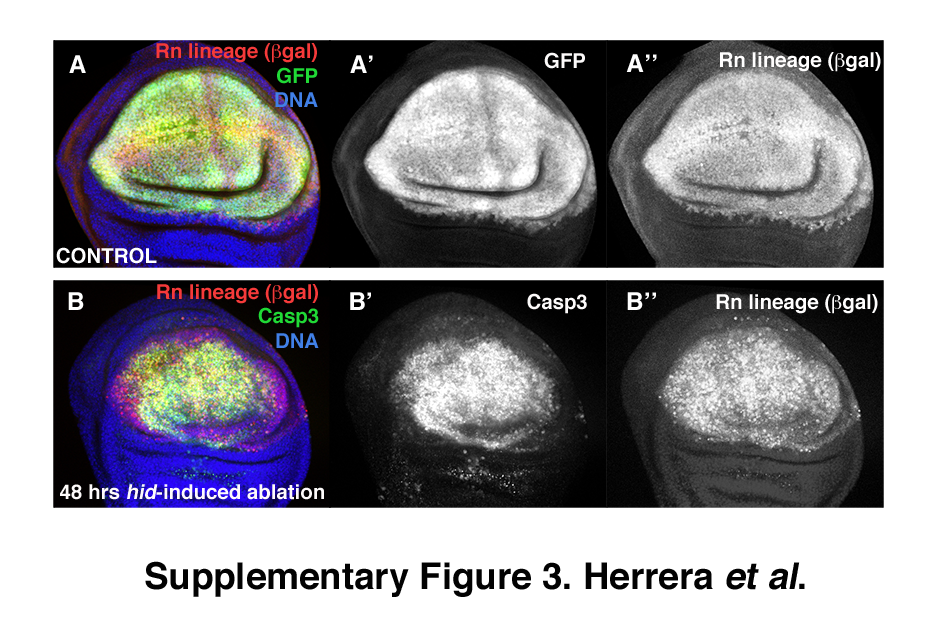

Supplement: Figure S3 — Method lo label all the cells of the Rn domain. Wing discs of genotype rn-Gal4 tub-Gal80TS UAS-GFP UAS-Flp act>stop>lacZ (A–A″, control) and rn-Gal4 tub-Gal80TS UAS-hid UAS-Flp act>stop>lacZ (B–B″, experimental) after 48 hrs of GFP (A–A″) or hid (B–B″) overexpression at 29°C. Note the lineage label in nearly 100% of rn-expressing cells (GFP-positive) in the control disc (A″) and the co-expression of apoptosis and lineage label in the ablated disc (B″). (TIF) [file pgen.1003446.s003.tif]

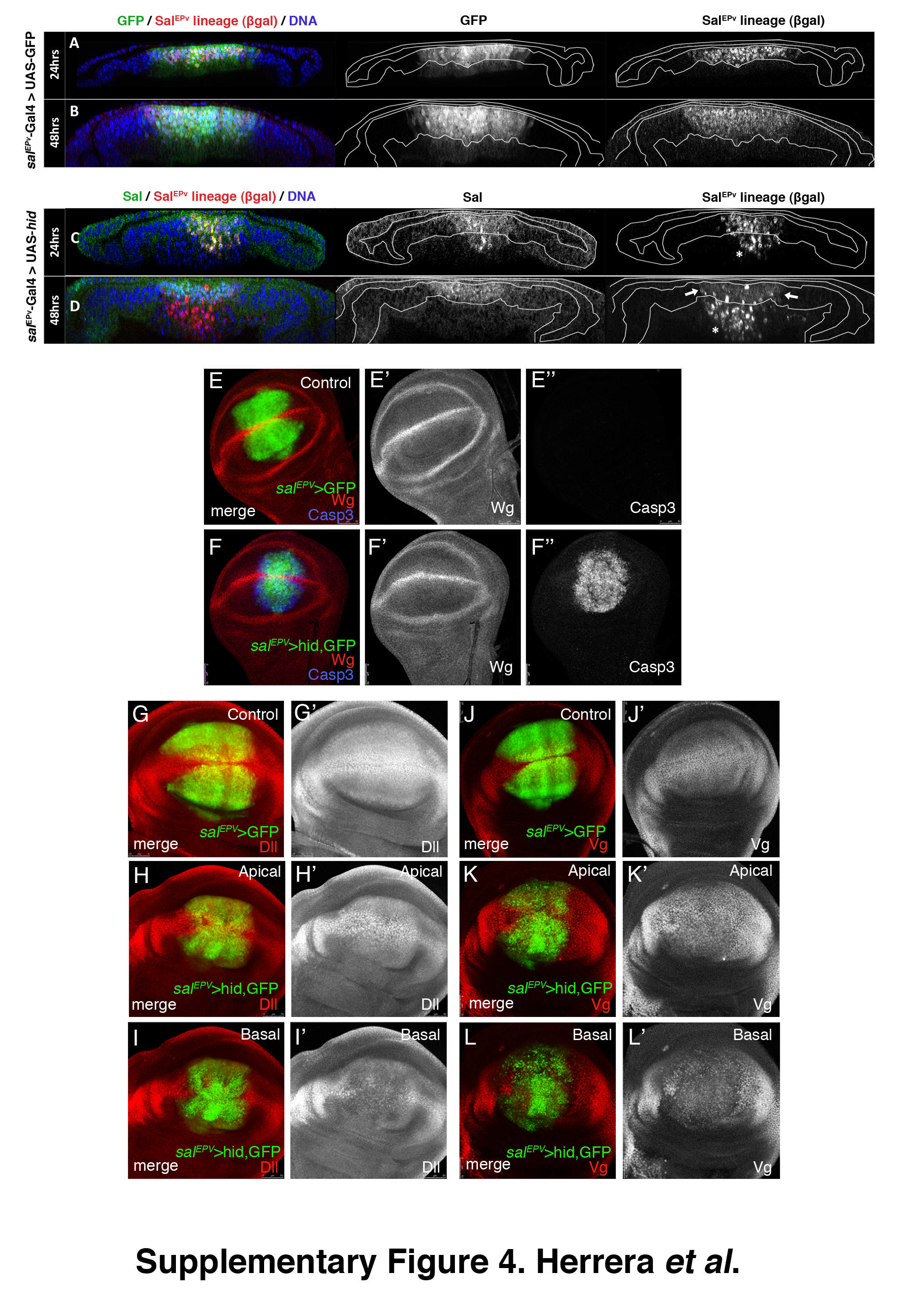

Supplement: Figure S4 — Ablation and reconstruction of the SalEPv domain. (A–D) Orthogonal sections perpendicular to the A/P border (at the point of maximum width of the SalEPv domain) of salEPv-Gal4 UAS-GFP/tub-Gal80TS; UAS-Flp act>stop>LacZ (control) (A–B) and salEPv-Gal4/UAS-hid tub-Gal80TS; UAS-Flp act>stop>LacZ (C–D) genotypes. Inductions times are indicated at the left, and the outline of the living epithelium (excluding apoptotic cells) is marked with a white line. Asterisks point to apoptotic cells which are being extruded basally (also visible as nuclear fragments). In (D), note that there are cells positive for Sal staining (green), which do not belong to the former Sal domain as they express low βgal activity, indicating de novo acquisition of Sal identity driven by a late recombination of the cassette. (E–E″) wg expression in non-ablating disc. (F–F″) Wg levels in ablated disc after 40 hrs of hid-expression in the SalEPv domain. (E, F) Merge and separate channels of Wg (E′. F′) and Caspase-3 (E″, F″) expression. Note that expression of wg is not altered. (G–I′) Dll expression (red) in control (G–G′) and apical (H. H′) or basal (I, I′) planes of ablated disc at 40 hrs of hid-induced ablation in the SalEPv domain. (J–L′) Vg expression (red) in control (J, J′) and apical (K, K′) or basal (L, L′) planes of ablated disc after 40 hrs of hid-induced ablation. The expression of both Dll and Vg in the apical plane remain essentially normal. (TIF) [file pgen.1003446.s004.tif]

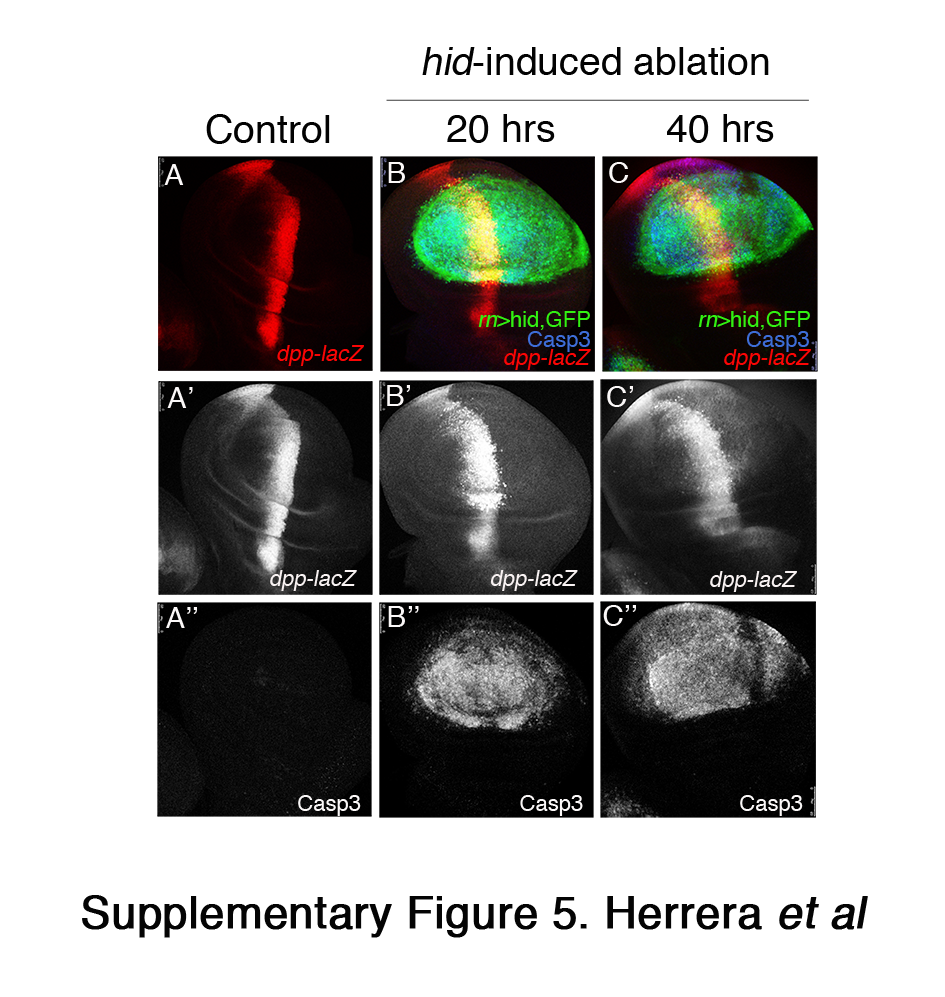

Supplement: Figure S5 — dpp expression after hid-induced ablation. (A–A″) dpp-lacZ expression in a control disc. (B–B″) dpp-lacZ expression in a rn>hid wing disc after 20 hrs of hid-induced ablation. (C–C″) dpp-lacZ expression in a rn>hid disc after 40 hrs of hid-induced ablation. dpp expression is label in red, the Rn domain in green and caspase activity in blue. Note that dpp expression remains normal during ablation even though Casp3 activity is very high. (A, B, C) Merges and separate channels of dpp-lacZ expression (A′, B′, C′) and Caspase-3 levels (A″, B″, C″). (TIF) [file pgen.1003446.s005.tif]

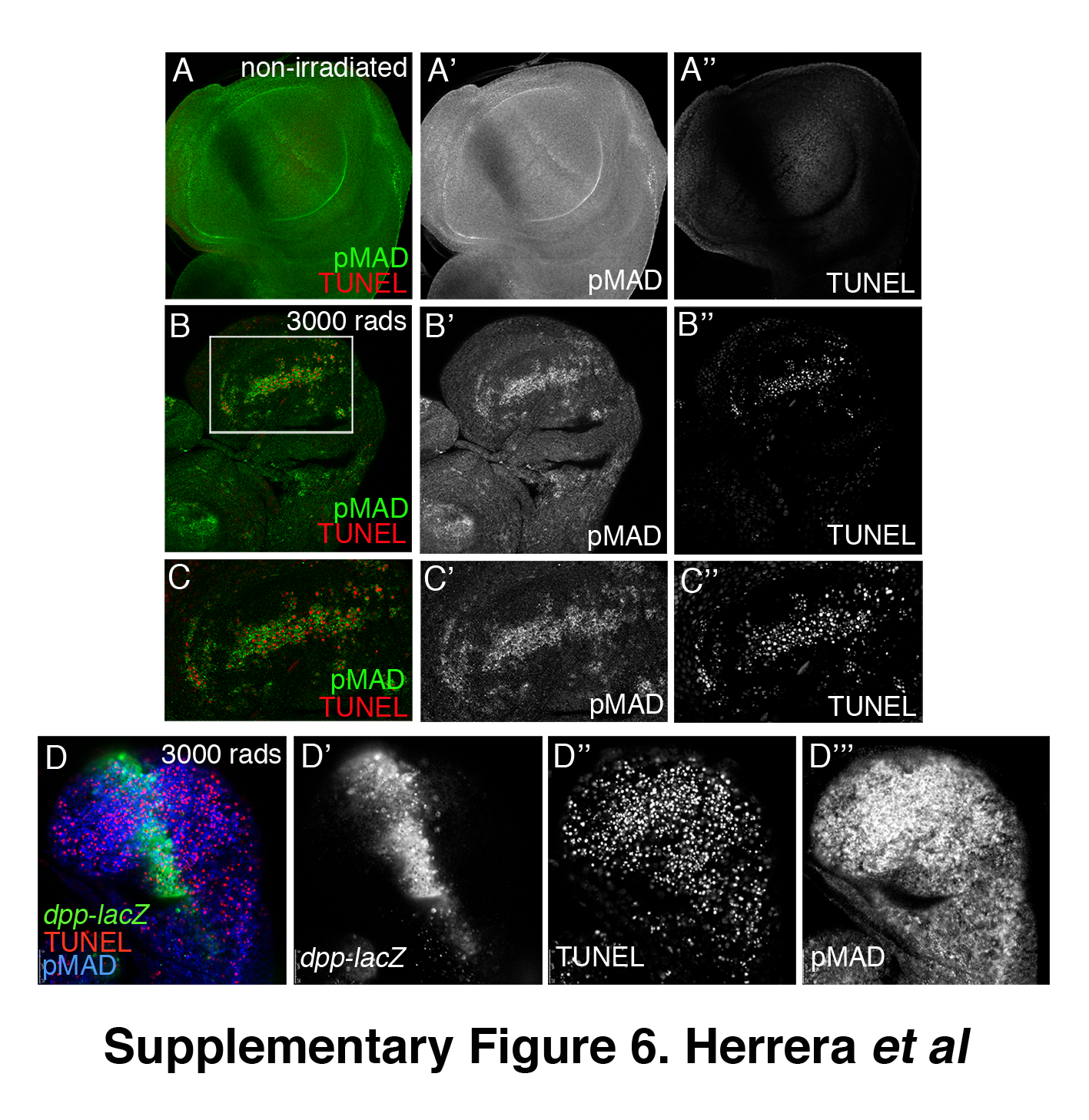

Supplement: Figure S6 — pMAD levels after massive irradiation. Third instar larvae were irradiated with 3000 rads and dissected 24 hrs after irradiation. Wing discs were extracted, fixed and immunostained as described in Experimental Procedures. (A–A″) pMAD expression at the A/P border in non-irradiated wing discs. (B–B″) pMAD expression localized in apoptotic cells (marked by TUNEL staining) in discs subjected to irradiation. (C–C″) Magnification of B–B″ images. Note the correspondence of TUNEL and pMad labels (D–D′″) Another disc showing massive apoptosis after irradiation. The high levels of pMAD correlate with TUNEL, but dpp expression remains normal. (TIF) [file pgen.1003446.s006.tif]

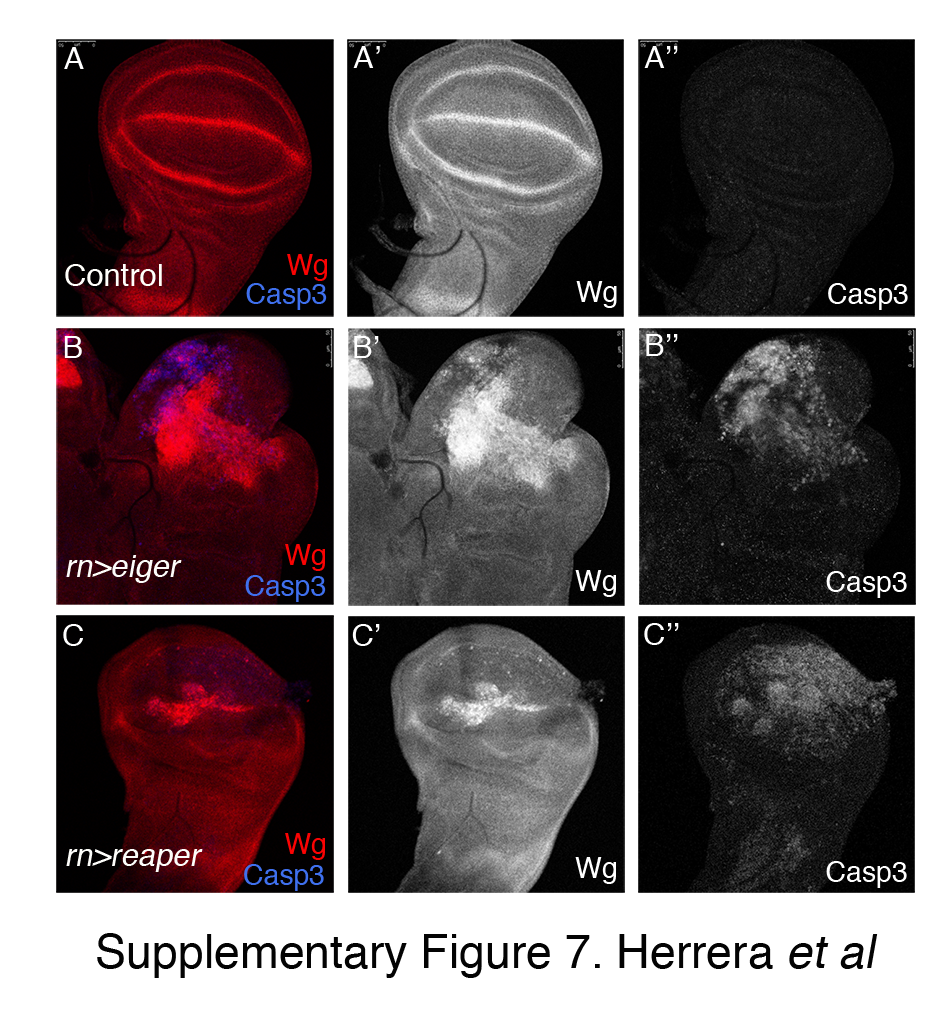

Supplement: Figure S7 — wg up regulation after eiger- or reaper-induced ablation. As previously reported [14], we observed a strong Wg up regulation in regenerating discs, after eiger overexpression in the Rotund domain. (A–A″) wg expression in control disc. (B–B″) wg up regulation in an rn>egr disc after 40 hrs of eiger expression. (C–C″) wg up regulation in an rn>rpr disc after 40 hrs of reaper expression. (TIF) [file pgen.1003446.s007.tif]
