# Supplementary material for: Tissue Homeostasis in the Wing Disc of Drosophila melanogaster: Immediate Response to Massive Damage during Development
Source: PLoS Genet. 2013 Apr 25;9(4):e1003446. doi: 10.1371/journal.pgen.1003446 (PMC3636033; doi:10.1371/journal.pgen.1003446)
Supplement: Table S1 — Summary of the effects induced by different pro-apoptotic vectors in the Rotund domain, as shown in Figures S2 and S7. In the row reporting the contact between the Peripodial and Columnar epithelia we distinguish between normal contact and lack of contact, which results in the appearance of a blister in the lumen. The rest is self explanatory. n.a. not analysed. (DOCX) [file pgen.1003446.s008.docx]

|  | **UAS-*hid*** | **UAS-*p53*** | **UAS-*diap1*-RNAi** | **UAS-*hep*^CA^** | **UAS-*egr*** | **UAS-*rpr*** |
| --- | --- | --- | --- | --- | --- | --- |
| Apoptotic levels | +++ | +++ | + | ++ | ++ | +++ |
| Epithelium continuity with Rn-positive cells | Yes | Yes | Yes | Yes | Yes | Yes |
| Apical integrity (Crumbs presence) | Yes | Yes | Yes | Yes | Yes | Yes |
| Tissue architecture after ablation | Normal | Normal | Normal | Disrupted. Extra foldings | Disrupted. Extra foldings | Disrupted. Extra foldings |
| Peripodial-Columnar epitheliums contact | In contact | In contact | In contact | Missing | Missing | Missing in late stages |
| Apoptotic debris extrusion | Basally | Basally | Basally | Apically | Apically + basally | Early basally.  Later apically + basally |
| Ectopic *wg* activation | no | n.a. | n.a. | n.a. | yes (+++) | yes (+) |
| Wing pouch reconstruction (after 48h of recovery) | full (+++) | n.a. | n.a. | n.a. | incomplete (+) | incomplete (+) |
